# Loss-of-function of the ciliopathy protein Cc2d2a disorganizes the vesicle fusion machinery at the periciliary membrane and indirectly affects Rab8-trafficking in zebrafish photoreceptors

**DOI:** 10.1371/journal.pgen.1007150

**Published:** 2017-12-27

**Authors:** Irene Ojeda Naharros, Matthias Gesemann, José M. Mateos, Gery Barmettler, Austin Forbes, Urs Ziegler, Stephan C. F. Neuhauss, Ruxandra Bachmann-Gagescu

**Affiliations:** 1 Institute of Molecular Life Sciences, University of Zurich, Zurich, Switzerland; 2 Center for Microscopy and Image Analysis, University of Zurich, Zurich, Switzerland; 3 Fred Hutchison Cancer Research Center, Seattle, Washington, United States of America; 4 Institute of Medical Genetics, University of Zurich, Schlieren, Switzerland; Medical College of Wisconsin, UNITED STATES

## Abstract

Ciliopathies are human disorders caused by dysfunction of primary cilia, ubiquitous organelles involved in transduction of environmental signals such as light sensation in photoreceptors. Concentration of signal detection proteins such as opsins in the ciliary membrane is achieved by RabGTPase-regulated polarized vesicle trafficking and by a selective barrier at the ciliary base, the transition zone (TZ). Dysfunction of the TZ protein CC2D2A causes Joubert/Meckel syndromes in humans and loss of ciliary protein localization in animal models, including opsins in retinal photoreceptors. The link between the TZ and upstream vesicle trafficking has been little explored to date. Moreover, the role of the small GTPase Rab8 in opsin-carrier vesicle (OCV) trafficking has been recently questioned in a mouse model. Using correlative light and electron microscopy and live imaging in zebrafish photoreceptors, we provide the first live characterization of Rab8-mediated trafficking in photoreceptors *in vivo*. Our results support a possibly redundant role for both Rab8a/b paralogs in OCV trafficking, based on co-localization of Rab8 and opsins in vesicular structures, and joint movement of Rab8-tagged particles with opsin. We further investigate the role of the TZ protein Cc2d2a in Rab8-mediated trafficking using *cc2d2a* zebrafish mutants and identify a requirement for Cc2d2a in the latest step of OCV trafficking, namely vesicle fusion. Progressive accumulation of opsin-containing vesicles in the apical portion of photoreceptors lacking Cc2d2a is caused by disorganization of the vesicle fusion machinery at the periciliary membrane with mislocalization and loss of the t-SNAREs SNAP25 and Syntaxin3 and of the exocyst component Exoc4. We further observe secondary defects on upstream Rab8-trafficking with cytoplasmic accumulation of Rab8. Taken together, our results support participation of Rab8 in OCV trafficking and identify a novel role for the TZ protein Cc2d2a in fusion of incoming ciliary-directed vesicles, through organization of the vesicle fusion machinery at the periciliary membrane.

## Author summary

Ciliopathies are human disorders caused by dysfunction of primary cilia, ubiquitous organelles involved in transduction of environmental signals to the cells. Concentration and regulation of signal detection proteins in the ciliary membrane is therefore tightly regulated through polarized vesicle trafficking and through a selective barrier at the ciliary base called the transition zone (TZ). Dysfunction of TZ proteins leads to human ciliopathies and to aberrant localization of ciliary proteins in animal models. In this work, we use zebrafish retinal photoreceptors as a model to explore the relationship between the TZ and upstream vesicle trafficking. Relying on modern technologies such as correlative light and electron microscopy and live imaging of fluorescently-tagged proteins, we identify a role for the TZ protein CC2D2A in organizing the components required for vesicle fusion at the periciliary membrane. We also characterize the movement dynamics of vesicles carrying light-detection proteins (opsins) towards the ciliary compartment of photoreceptors *in vivo* and provide novel data in support of the recently questioned involvement of the small GTPase Rab8 in opsin-carrier vesicle trafficking in photoreceptors.

## Introduction

Ciliopathies are an expanding group of human disorders caused by primary cilium dysfunction and unified by a wide array of overlapping phenotypes: cystic kidneys, central nervous system malformations and retinal degeneration among others [1–3]. Primary cilia are ubiquitous organelles that consist of a mother centriole-derived basal body (BB) and a microtubule-based axoneme ensheathed by a specialized membrane. Primary cilia are involved in transduction of a variety of environmental signals to the cell, including morphogens and light. To serve this purpose, the ciliary membrane is enriched with specific receptors and channels required for signal detection [4]. As cilia are devoid of protein synthesis machinery [5], ciliary membrane compartmentalization is achieved by highly controlled polarized vesicle trafficking subject to RabGTPase regulation [6] and by a selective barrier at the base of the cilium likely formed by the transition zone (TZ) [7]. Mutations in several genes encoding TZ proteins lead to Joubert syndrome (JBTS; OMIM: 213300), a representative ciliopathy characterized by a very specific cerebellar malformation–the molar tooth sign–and associated in 30% of cases with retinal involvement due to photoreceptor (PR) dysfunction [8].

Retinal involvement is common in ciliopathies since the outer segment (OS) of PRs, which is the site of the phototransduction cascade, is a highly specialized primary cilium [9]. OSs consist of stacks of membranous disks organized around a microtubule-based axoneme. These membranous disks contain the proteins required for phototransduction, such as the photopigment opsin [10]. Trafficking of opsins towards the OSs is thought to be controlled by the small GTPase Rab8, as dominant negative GDP-locked Rab8 expression in frog PRs leads to opsin-carrier vesicle (OCV) accumulation at the base of the PR OSs [11, 12]. Further work in frog PRs identified a complex initiated by Arf4 in the trans-Golgi network including ASAP1, Rab11, FIP3, and subsequently Rabin8 and Rab8. This complex was shown to be involved in opsin delivery to the ciliary compartment [13, 14]. In addition, Rab11 and Rabin8, the guanidine exchange factor for Rab8, were shown to promote ciliary membrane biogenesis in RPE-hTERT cells [15–17]. While two RAB8 paralogs exist in humans (RAB8A and RAB8B), which were both found to be involved in ciliogenesis in RPE cells [18], it remains undefined which paralog is important in OCV trafficking in PRs. In addition, work on *Rab8* knock-out mice questioned the importance of this GTPase in ciliogenesis. Indeed, double *rab8a;rab8b* knock-out mice revealed mislocalization of apical proteins in intestinal cells but required additional knock-down of the close Rab8-relative Rab10 to develop ciliogenesis defects in fibroblasts [19]. This work suggested that various Rabs can compensate for loss of function of each other. However, a mouse *rab8a* knock-out displayed no retinal phenotype despite additional expression of dominant negative forms of Rab8b, Rab11a, Rab11b and Rab10, questioning the role of Rab8 in OCV trafficking in PRs [20].

Once OCVs have reached their target membrane, vesicle fusion is thought to be mediated by the Exocyst and SNARE (soluble N-ethylmaleimide-sensitive factor attachment protein receptor) proteins. SNAREs present on the vesicle surface (v-SNAREs) and on the target membrane (t-SNAREs) pair up and work as catalysts to provide the mechanical force required to mediate membrane fusion [21]. The Exocyst is a multisubunit protein complex implicated in tethering of secretory vesicles to the plasma membrane in several exocytosis processes including ciliogenesis [22, 23]. The Exocyst localizes at the ciliary base in cell culture [24] and in the ciliary stalk of PRs in frog retina [25]. Moreover one of its components, Sec15, has been found to interact directly with Rab8 [22]. Interestingly, the Exocyst components Sec8, Sec10 and Exo84 have been involved in the pathogenicity of various ciliopathies, including Joubert syndrome and the related Meckel syndrome [26–30].

Joubert syndrome is a ciliopathy caused by mutations in one of at least 30 genes, many of which encode TZ proteins interacting with each other in large multi-protein complexes. Dysfunction of CC2D2A, one such TZ protein, is one of the most common causes for JBTS, accounting for ~10% of JBTS families [8]. As for other TZ proteins, its dysfunction leads to loss of ciliary protein localization in mice [31]. Similarly, we have previously reported intracellular opsin mislocalization and massive vesicle accumulation in PRs of the zebrafish *cc2d2a* ^*uw38*^ mutant, suggesting the involvement of Cc2d2a in ciliary trafficking and supporting the role of the TZ in ciliary protein content regulation [32, 33]. While we also observed loss of Rab8 puncta in the absence of Cc2d2a function in this model, the link between the TZ protein Cc2d2a, the small GTPase Rab8 and ciliary-directed trafficking remained unexplored.

In this study, we show that Rab8-coated opsin-containing vesicles accumulate progressively at the apical portion of *cc2d2a-/-* PRs as a result of a vesicle fusion defect. Using correlative light and electron microscopy (CLEM) as well as live imaging in zebrafish PRs, we provide a detailed characterization of Rab8-mediated trafficking in this cell type. Our findings support a role for Rab8 in OCV trafficking and demonstrate redundancy between Rab8 paralogs in both rods and cones of wild-type fish. Time-lapse analysis of Rab8-tagged particles in *cc2d2a*-/- PRs reveals, besides partial mislocalization of Rab8 to the cytoplasm, no substantial difference in movement kinetics for remaining vesicular-associated Rab8 particles compared to wild-type PRs, suggesting that the observed OCV accumulation is not caused by a direct defect in trafficking but rather by deficient vesicle fusion. In support of this model, we observe loss of elements of the vesicle fusion/tethering machinery normally present at the apical membrane of PRs such as t-SNAREs SNAP25 and Syntaxin3 and the Exocyst component Exoc4/Sec8 consequent to Cc2d2a loss-of-function. Together, these results indicate that Cc2d2a plays a crucial role in organization of the vesicular docking sites at the periciliary membrane, allowing OCVs to fuse and deliver their cargo at the base of the OSs.

## Results

### Vesicles accumulate progressively from the onset of outer segment formation in the apical portion of *cc2d2a*-/- photoreceptors

**Fig 1:**
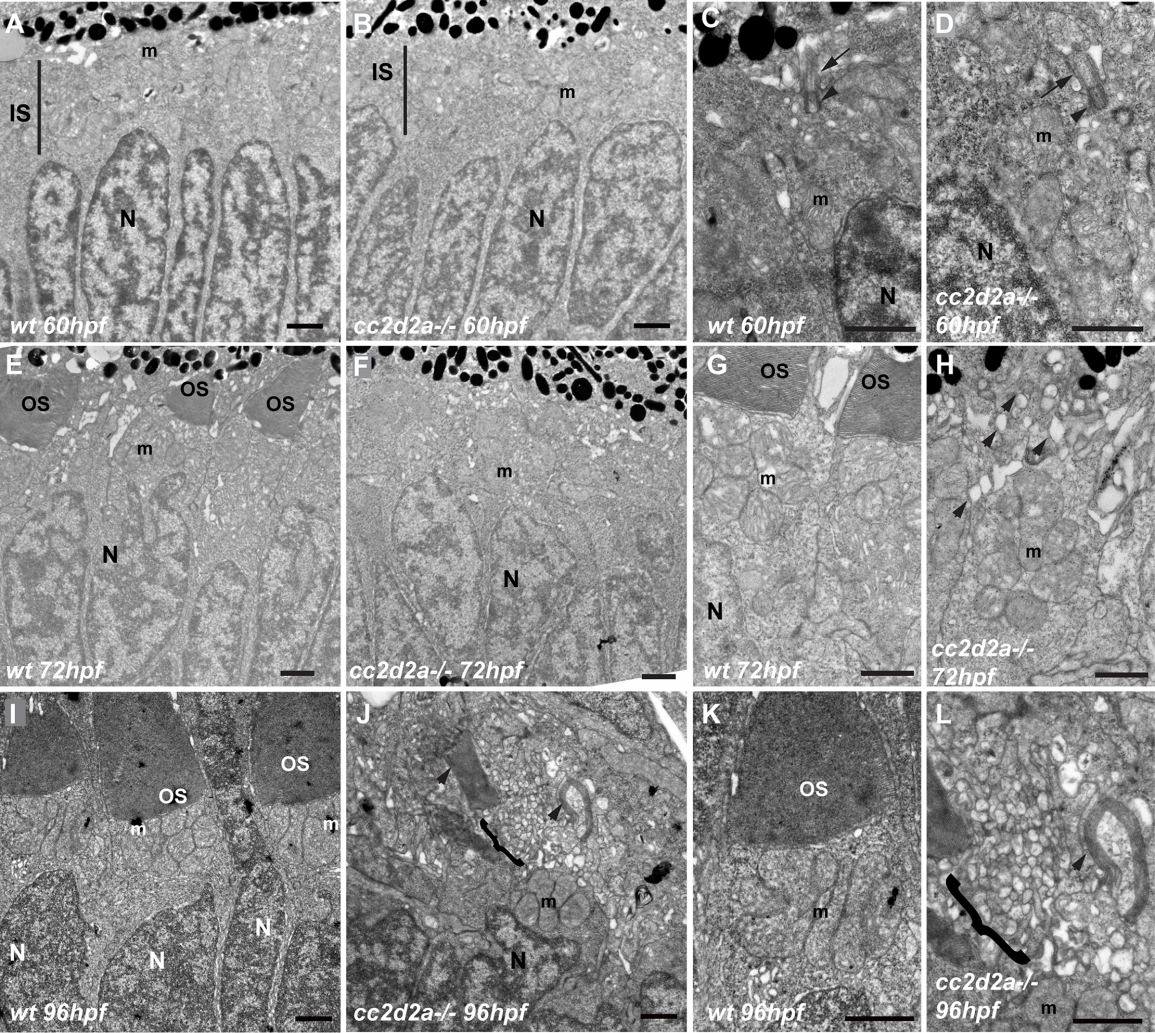
**Vesicle accumulation is progressive from the onset of OS formation in *cc2d2a-/-* PRs (A-D)** Transmission electron microscopy images (TEM) of retinal sections at 60 hpf are indistinguishable between wild-type (wt) **(A, C)** and *cc2d2a-/-* **(B, D)**, including with respect to basal body docking (arrowheads in **C-D**) and extension of the connecting cilium (arrows in **C-D**). **(E-H)** Retinal sections at 72 hpf: note the nascent outer segments (OS) in wt **(E, G)**, but the quasi-absence of OSs in *cc2d2a-/-* with onset of apical accumulation of vesicular structures **(F, H**, arrowheads). **(I-L)** Retinal sections at 96 hpf: well-formed OSs are present apical to the mitochondrial cluster in wt **(I, K)**, while in mutant photoreceptors an increased number of vesicles (brackets) are found in the apical portion of the cell together with misshapen membrane stacks (arrowheads) instead of OSs **(J, L).** Scale bars are 1 μm in all panels. *m* mitochondria, *N* nuclei, *OS* outer segments, *wt* wild-type, *hpf* hours post fertilization.

Our previous studies of the *cc2d2a*^*uw38*^ zebrafish null mutant identified massive accumulation of vesicular structures in the apical portion of zebrafish photoreceptors (PRs) co-existing with misshapen outer segments (OSs) at 5 days post fertilization (dpf) [33]. To rule out the possibility that these structures are the result of a degenerative process, we first performed a TUNEL assay to assess PR cell death, which we found to be minimally increased compared to wild-type at 3 dpf only and not increased at 4 dpf (S1A–S1B” and S1F Fig). Furthermore, the morphology of PR nuclei was not affected in *cc2d2a*-/- retinae, in contrast to what is observed in the degenerating retina of the well-studied ciliary zebrafish mutant *ift88-/-/oval* [34] (S1C–S1E Fig). We further performed transmission electron microscopy (TEM) to analyze the ultrastructure of *cc2d2a* mutant retinae at earlier time points spanning OS formation. At 60 hpf, wild-type (wt) PRs have taken on their typical cell morphology including an apico-basally elongated nucleus, an inner segment containing the forming mitochondrial cluster (Fig 1A) and a basal body (BB) that has already docked to the apical membrane with extension of the connecting cilium (Fig 1C). At this stage, *cc2d2a-/-* PRs are morphologically indistinguishable from wt PRs (Fig 1B) including in particular docking of the BB to the apical membrane and extension of the connecting cilium (Fig 1D and S2 Fig). However, at later stages we observe that ciliary membrane stacking is impaired in *cc2d2a*-/- PRs. Nascent OSs are observed in a majority of wt PRs at 72 hpf (Fig 1E and 1G) but are mostly absent in *cc2d2a*-/- retinae where vesicular structures begin to accumulate instead (Fig 1F and 1H). While wt PRs extend OSs of increasing length at 4 dpf (Fig 1I and 1K), vesicular accumulation increases progressively over time in *cc2d2a*-/- PRs (Fig 1J and 1L) becoming massive at 5 dpf, as previously reported [33]. Therefore, we conclude that accumulation of vesicles is progressive from onset of OS formation and not the result of a degenerative process.

### Accumulated vesicles are opsin-carrier vesicles

**Fig 2:**
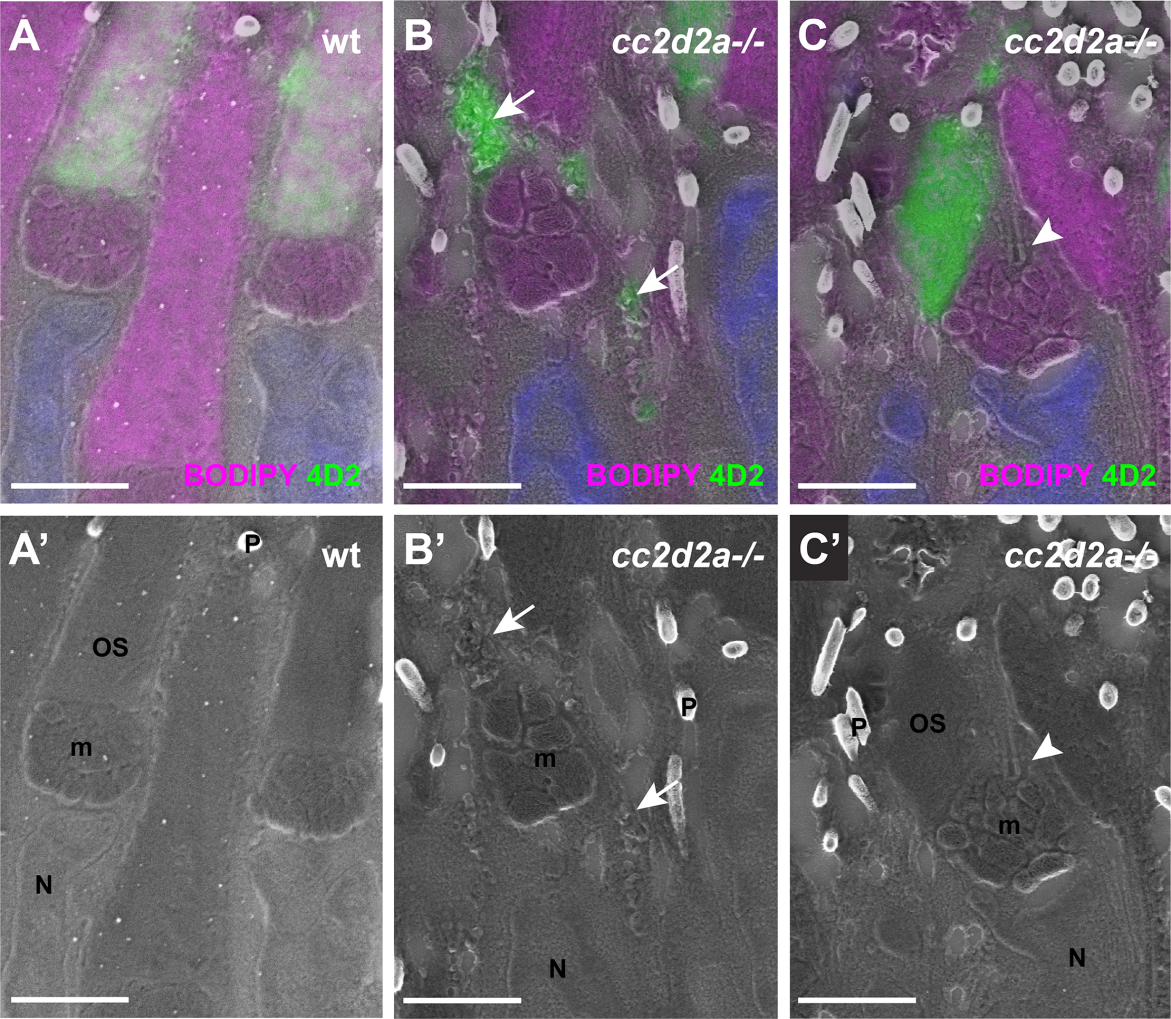
**Accumulated vesicles in *cc2d2a-/-* PRs are opsin-carrier-vesicles (A-C)** 5 dpf correlative light and electron microscopy (CLEM) image of retinal sections stained with BODIPY (magenta) to mark membranes of the outer segment and the mitochondrial cluster and with 4D2 (green) to label rhodopsin and red-green cone opsin. **(A’-C’)** are the corresponding scanning electron microscopy (SEM) images. Note that while 4D2 staining only localizes at the outer segments of wild-type (wt) PRs **(A)**, it is visible in accumulated vesicles in *cc2d2a-/-* PRs **(B,** arrows) and in dysmorphic outer segments **(C)**. Also note normal cilium docking in *cc2d2a-/-* PRs **(C-C’,** arrowhead). Scale bars are 2 μm in all panels. *m* mitochondria, *N* nuclei, *OS* outer segments, *P* pigment in melanosomes, *wt* wild-type.

To ensure that these accumulated membranous structures are opsin-carrier vesicles (OCVs) and thus were ciliary targeted, we used a recently developed correlative light and electron microscopy (CLEM) method [35, 36]. This method enables us to overlay immunodetected opsin on ultrathin sections with scanning electron microscopy (SEM) images to determine the precise ultrastructural localization of the opsins. Structures of interest such as OSs (Fig 2A–2A’), accumulated vesicles (Fig 2B–2B’, arrows) or cilia (Fig 2C–2C’, arrowhead) are easily recognizable on the SEM images. Using the 4D2 antibody, which recognizes rhod- and red-green cone opsin, we found opsin signal in OSs of wild-type PRs (Fig 2A). In *cc2d2a*-/- PRs, opsins localize to aberrant membrane stacks of dysmorphic OSs as well as to the accumulated vesicles at 5 dpf (Fig 2B and 2C). Similar opsin-positive vesicle accumulation was observed already at 3 dpf with onset of aberrant membrane stacking (S3 Fig). In comparison, transport of transmembrane proteins such as Cacna1fa to other cellular compartments than the OS (synapse) is unaffected in *cc2d2a-/-* PRs (S4 Fig). Collectively, our data indicate that OCVs accumulate progressively in *cc2d2a*-/- PRs as a result of either a ciliary-specific trafficking or a vesicle fusion defect.

### The small GTPase Rab8 associates with opsin-carrier vesicles

Our previous study of the zebrafish *cc2d2a*^*uw38*^ mutant described mislocalization of the small GTPase Rab8 in PRs lacking Cc2d2a [33]. This suggested that a defect in Rab8-mediated trafficking could underlie the observed vesicle accumulation, given the ascribed roles for Rab8 in regulation of polarized vesicle trafficking to cilia [15, 16, 18] and in delivery of opsin-carrier-vesicles (OCVs) in PRs [14]. However, recent work has questioned this role for Rab8 paralogs in OCV transport in mouse [20]. To elucidate whether Rab8 participates in opsin transport in zebrafish and to investigate the consequences of Cc2d2a loss-of-function on Rab8-mediated trafficking, we generated transgenic fish lines stably expressing tagged Rab8 in PRs.

Zebrafish have 3 Rab8 paralogs: Rab8a, Rab8b and Rab8b-like. Based on synteny and sequence similarity (93.2% identity at the amino acid level), zebrafish Rab8a (NM_001089562) appears to be the true ortholog of human RAB8A (NM_005370). The chromosomal region around the human RAB8B gene (NM_016530, chromosome 15) shows conserved synteny with two zebrafish genomic regions, on chromosome 7 (Rab8b, NM_001099259) and on chromosome 25 (Rab8b-like, XM_021470743.1), suggesting that these are zebrafish paralogs resulting from the teleost-specific genome duplication (S5 Fig). Consequently, we now call Rab8b-like Rab8ba and Rab8b Rab8bb (S6 Fig). In any case, all three zebrafish Rab8 paralogs share very high sequence similarity with each other and with the human RAB8A and B paralogs (S7 Fig).

**Fig 3:**
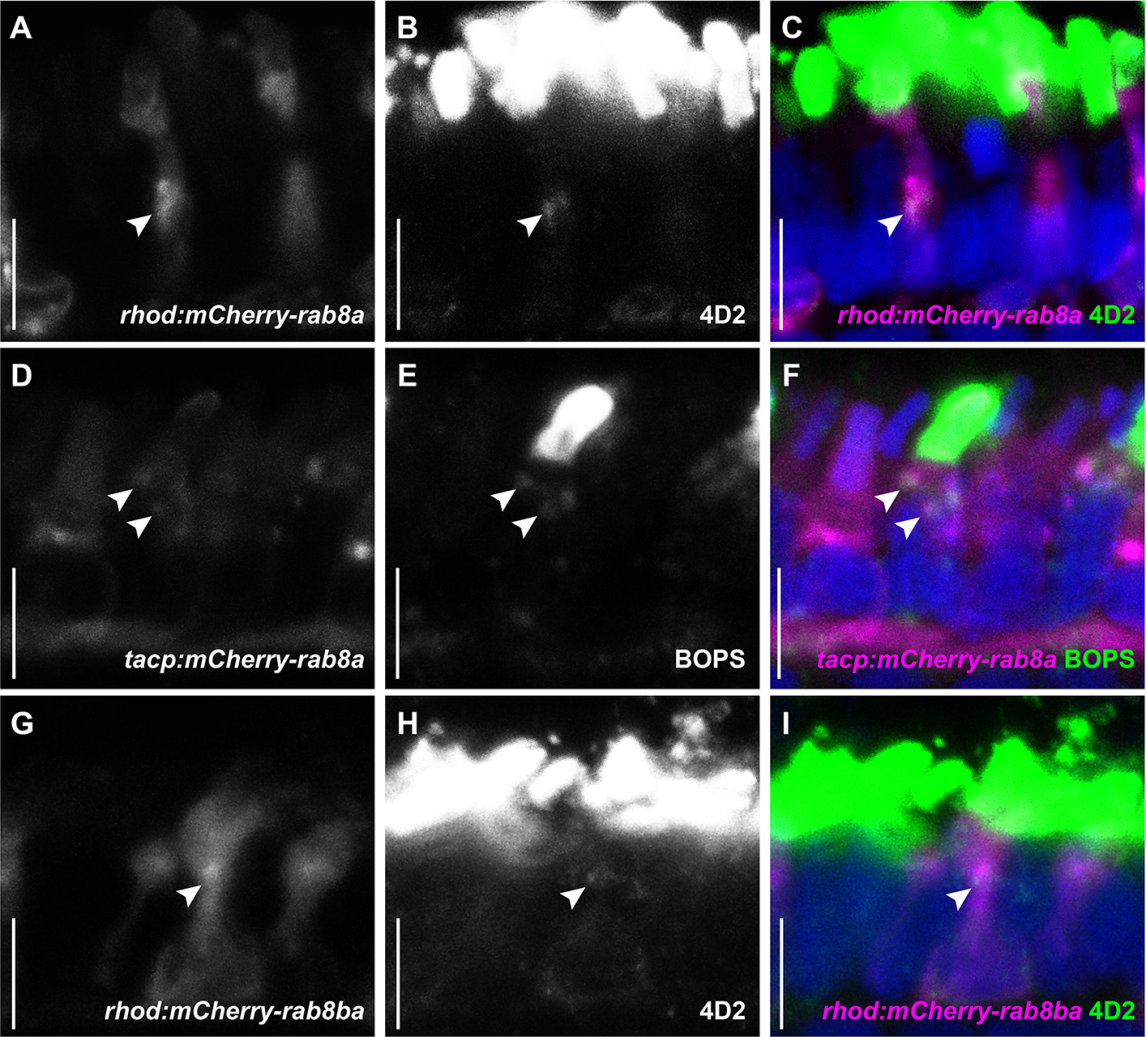
**Rab8a and Rab8ba co-localize with endogenous opsins (A-C)** 5 dpf cryosections of wild-type (wt) zebrafish expressing mCherry-tagged Rab8a in rods (magenta), stained with the anti-opsin antibody 4D2 (green). **(D-F)** 5 dpf cryosections of wt zebrafish expressing mCherry-tagged Rab8a in cones (magenta), stained with anti-blue opsin (green). **(G-I)** 5 dpf cryosections of wt zebrafish expressing mCherry-tagged Rab8ba in rods (magenta), stained with the anti-opsin antibody 4D2 (green). Arrowheads indicate examples of co-localization in all cases. Scale bars are 5 μm in all panels.

**Fig 4:**
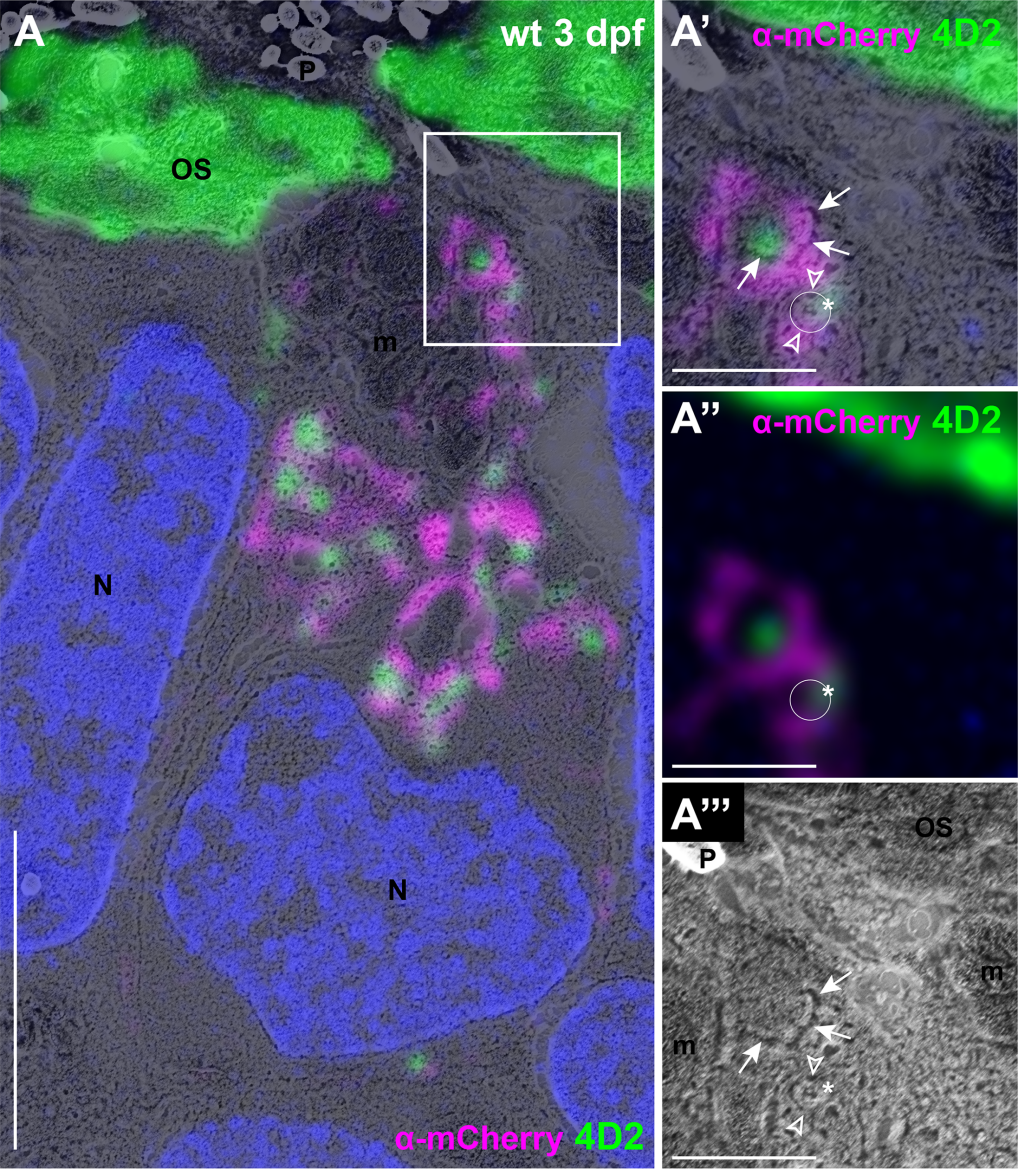
**Rab8 and opsin are associated with membrane-bound vesicular structures (A)** CLEM image of wild-type (wt) transgenic zebrafish at 3 dpf expressing mCherry-tagged Rab8a in rods stained with anti-mCherry (magenta) and anti-opsin antibody 4D2 (green).**(A’)** High magnification image of the boxed area in (A) showing a clearly individualizable small round membrane-bound structure (white circle) coated with Rab8a signal on either side (empty arrowheads over the magenta signal) and with opsin signal over the edge of the structure (asterisk over the 4D2 signal), compatible with transmembrane opsins in a Rab8-coated vesicle. **(A”)** Immunohistochemistry image only from (A’). The white circle is placed where the vesicular structure is observed in the SEM image. **(A”‘)** SEM image only from (A’), showing the vesicular structure. The empty arrowheads are placed where the Rab8a signal is observed and the asterisk is located over the opsin signal. The additional m-Cherry and opsin signal likely represent a conglomerate of several vesicular structures with multilobulated membranes (arrows in A’ and A”‘). Scale bars: 4 μm in **A** and 1 μm in **A’-A”‘**. *m* mitochondria, *N* nuclei, *OS* outer segments, *P* pigment, *wt* wild-type.

To characterize Rab8-directed trafficking in zebrafish PRs and determine whether different Rab8 paralogs could play a role in OCV trafficking, we chose to use the zebrafish Rab8a and Rab8ba sequences. We generated lines expressing N-terminal mCherry-tagged Rab8a in rods (*tg(rhod*:*mCherry-rab8a)*) and cones (*tg(tacp*:*mCherry-rab8a)*) as well as Rab8ba in rods (*tg(rhod*:*mCherry-rab8ba)*). To determine if the different Rab8 paralogs co-localized with opsin-carrier vesicles, we first performed immunofluorescence using the 4D2 antibody (recognizing rhodopsin and red-green cone opsin) on retinal sections of transgenic animals expressing either mCherry-Rab8a or mCherry-Rab8ba. In both cases, we observed co-localization of mCherry-Rab8 with endogenous opsins in both cones and rods (Fig 3A–3I), whereby ~ 50% of opsin puncta co-localized with Rab8a/Rab8ba (48.8%±27.8% for Rab8a and 50.2%±21.9% for Rab8ba, n > 50 PRs from 11 animals per condition; the remaining opsin-positive puncta could be co-localizing with either endogenous Rab8 paralog or with other Rabs). We further transiently co-expressed heat-shock inducible GFP-tagged rhodopsin (*hsp70*:*rhod-GFP*) [37] in Rab8 transgenic fish to obtain time-lapse videos in live 5 dpf larvae and observed co-movement of Rhodopsin-GFP particles with mCherry-Rab8 particles *in vivo* (S1 video). Finally, using CLEM, we found that opsin signal is associated with mCherry-Rab8a in vesicular-like structures (Fig 4). Together, these results confirm that the small GTPase Rab8 is present at the surface of OCVs in both cones and rods and suggest possible redundancy between Rab8 paralogs in the transport of opsins.

### mCherry-Rab8a partially mislocalizes in *cc2d2a*-/- cones

**Fig 5:**
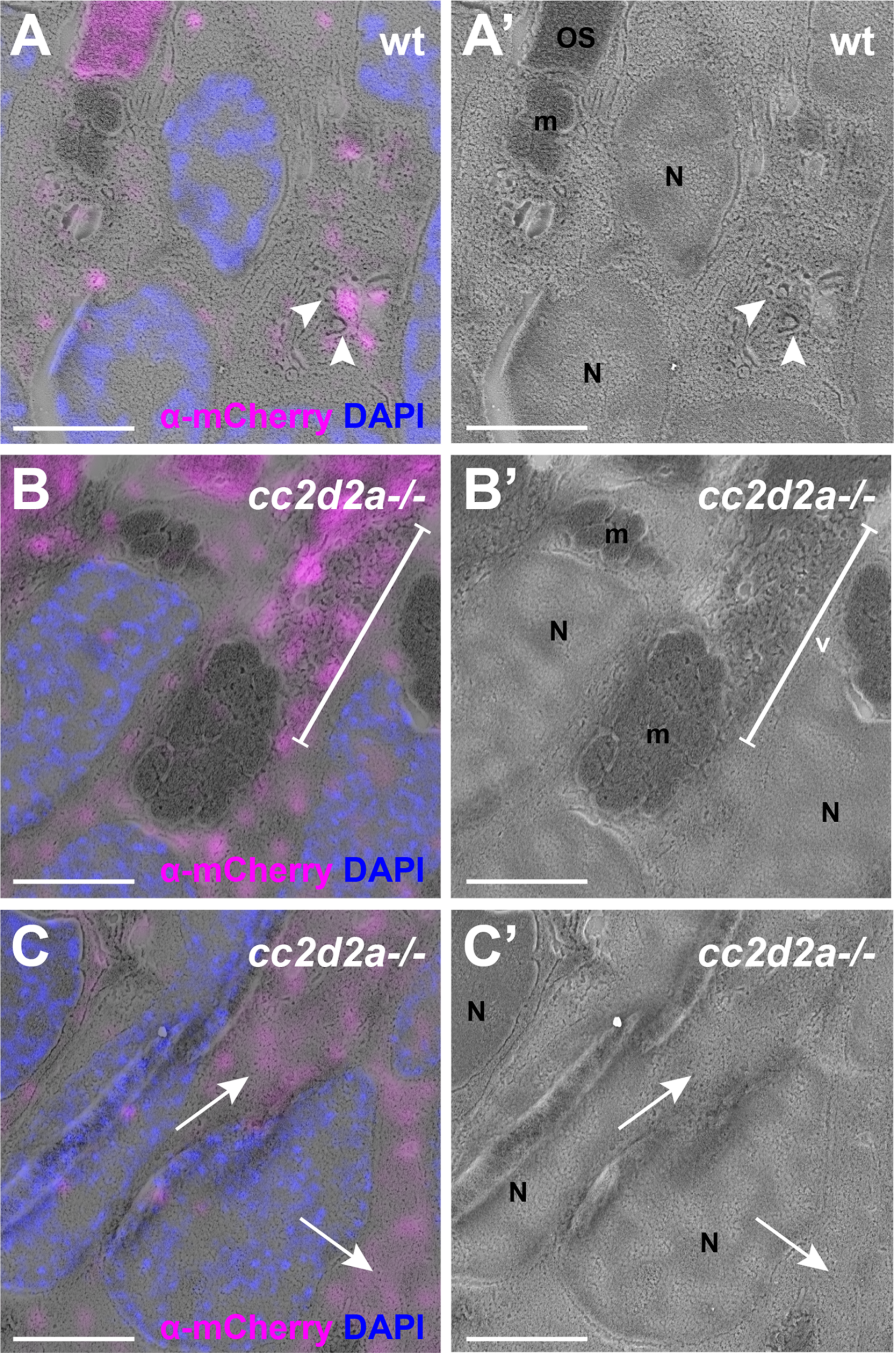
**Rab8a partially mislocalizes in *cc2d2a-/-* cones** 5 dpf CLEM images of wild-type (wt) **(A-A’)** and *cc2d2a*-/- **(B-C’)** zebrafish expressing mCherry-tagged Rab8a in cones *(tg(tacp*:*mCherry-rab8a)*, magenta). The mCherry signal is enhanced with anti-mCherry antibody. **(A-A’)** Note the localization of Rab8a (magenta) to vesicular structures (arrowheads) in wt. **(B-B’)** In *cc2d2a*-/- cones, Rab8a (magenta) localizes to accumulated vesicles (v, bracket) as well as to non-membrane-delimited cytoplasmic areas **(**arrows in **C**-**C’)**. Scale bars are 3 μm in all panels. *OS* outer segment, *m* mitochondria, *N* nuclei, *v* vesicular structures, *wt* wild-type.

Our previous work suggested Cc2d2a to be required for the punctate localization of Rab8, as transiently expressed mCherry-Rab8 was diffusely localized in a majority of *cc2d2a-/-* cone PRs, while only a subset of mutant PRs maintained punctate Rab8 expression [33]. We now confirmed these observations in transgenic lines stably expressing mCherry-Rab8. In wild-type PRs, mCherry-Rab8 signal was present as puncta, predominantly in the inner segment (IS), but also to a lesser extent more basally in the PRs, with no clear signal inside the OSs. Immunostaining using an anti-Rab8 antibody confirmed that these mCherry-puncta contain Rab8 (S8A–S8F Fig). In non-transgenic retinae, the signal provided by the anti-Rab8 antibody was less strong than observed with the transgene but showed similar puncta in the IS which were also positive for opsins, indicating that endogenous Rab8 has a similar subcellular localization pattern as observed with the transgene (S8G–S8I Fig). To further evaluate the localization of Rab8 at the ultrastructural level, we performed CLEM on mCherry-Rab8 expressing eyes, which required enhancement of the mCherry signal with an anti-mCherry antibody. In wt PRs, we observed mCherry-positive puncta that localized to membrane-delimited vesicular structures (Fig 5A–5A’, arrowheads). In *cc2d2a-/-* PRs, mCherry signal was localized to accumulated vesicles (Fig 5B–5B’ bracket). In addition, we observed a weaker diffuse signal in the cytoplasm of mutant PRs, not delimited by membrane (Fig 5C–5C’, arrows). Of note, mCherry signal in OSs (Fig 5A) became apparent only after antibody-enhancement of the signal, and was absent from non-enhanced transgenic retinae (S8A and S8D Fig) or from non-transgenic retinae stained with anti-Rab8 antibody (S8H–S8I Fig), suggesting either non-specific OS antibody signal or very low-grade OS expression of transgenic mCherry-Rab8 due to overexpression and visible only after enhancement. Collectively, these data indicate that Rab8 (both endogenous and mCherry-tagged) localizes to puncta representing vesicular structures and that loss of Cc2d2a function leads to partial mislocalization of Rab8 to the cytoplasmic compartment, while Rab8-coated vesicles also remain present in *cc2d2a* mutant PRs.

### Live imaging of Rab8-mediated trafficking shows similar kinetics in rods and cones and suggests redundant functions between Rab8 paralogs

**Fig 6:**
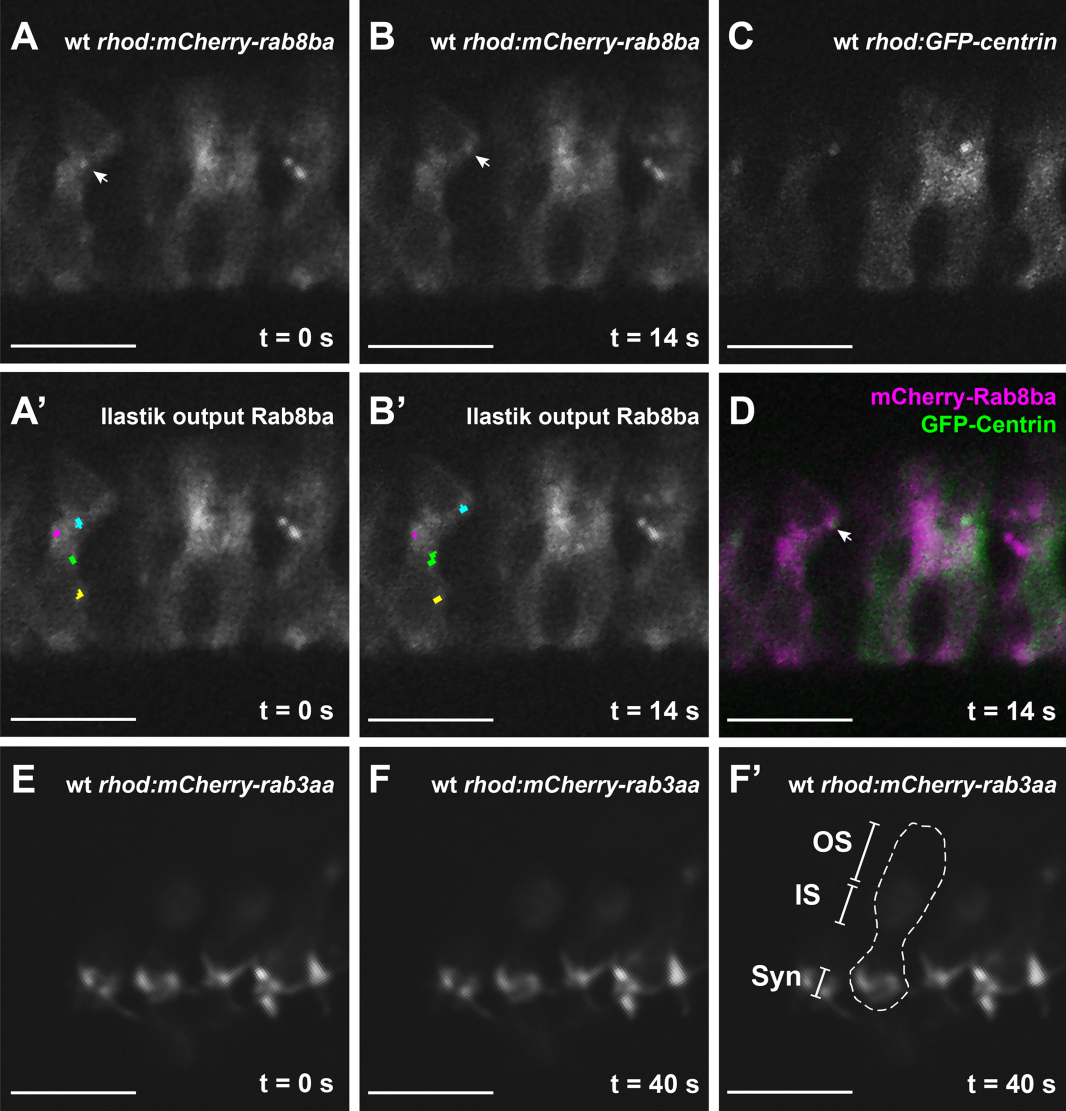
**Rab8-particles display dynamic movement patterns and transiently approach the BB (A-B)** Time-lapse imaging of a 5 dpf wt zebrafish retina expressing Rab8ba in rods (*tg(rhod*:*mCherry-rab8ba*)). The Rab8 particle marked with an arrow in all time frames can be recognized and followed by the Ilastik tracking software (cyan overlay) **(A’-B’)**. This particle transiently approaches the GFP-tagged basal body (**C** and merge in **D**). The movement is specific for Rab8 as transiently expressed Rab3aa in rods *(rhod*:*mCherry-rab3aa)* **(E-F’)** exhibits the expected synaptic localization **(F’)**. Scale bars are 10 μm in all images. *OS* outer segment, *IS* inner segment, *Syn* Synapse.

Taking advantage of the stable transgenic lines expressing mCherry-tagged Rab8 paralogs in rods and cones, we went on to characterize Rab8-trafficking in PRs *in vivo* in a whole tissue context. For that purpose, we obtained time-lapse videos of live 5 dpf old transgenic larvae stably co-expressing mCherry-Rab8 with GFP-hCentrin, the latter serving as an immobile cellular reference to label the basal body (BB) (S2 video). We found Rab8-tagged puncta to display a complex apico-basal shuffling movement, mostly in the inner segment but also spanning the entire length of the PR from the BB to the synapse, with a subset of puncta approaching the BB (Fig 6A–6D). To ensure that this movement was not an artifact driven by the overexpression of Rab8, we transiently overexpressed mCherry-Rab3aa and analyzed its behavior via live imaging. mCherry-Rab3aa strongly localizes at the PR synapse as predicted [38], where it shows predominantly localized movement in the synaptic region over the 10 minute duration of the time-lapse (S3 video and Fig 6E and 6F’), indicating that overexpressed Rabs exhibit the predicted endogenous behavior. Similarly, previous work in zebrafish using overexpressed fluorescently-tagged Rab5, Rab7 and Rab11 showed distinct localization patterns for the different Rabs consistent with the predicted endosomal compartments for each Rab and a live lipophilic dye uptake assay supported the biological relevance of such overexpression assays [39]. Furthermore, in our assay, despite some increase in vesiculo-tubular structures in a subset of PRs (S9 Fig), overexpression of Rab8 did not substantially affect retinal ultrastructure in the transgenic animals at 5 dpf.

**Fig 7:**
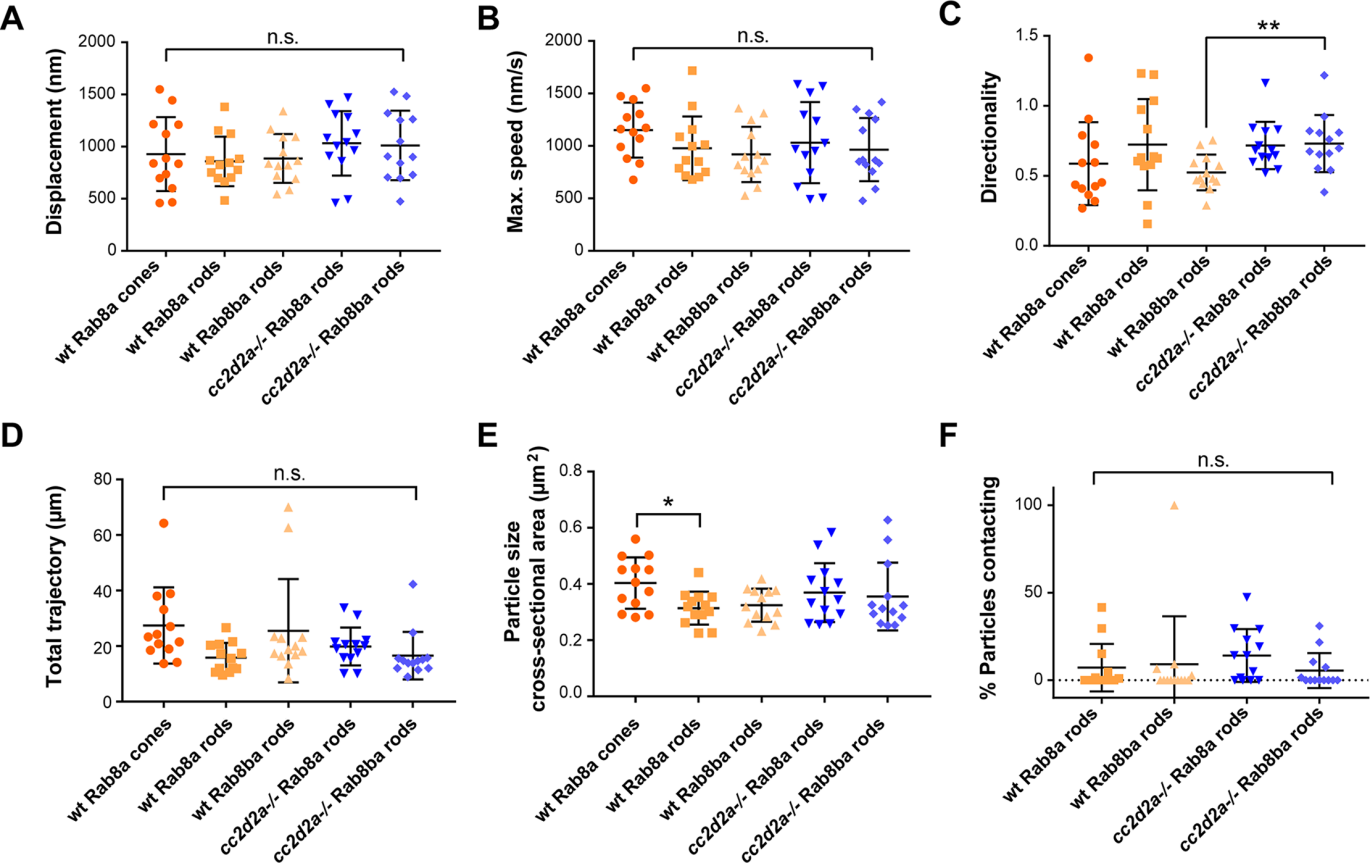
**Rab8-trafficking kinetics are conserved between different paralogs in wt rods and cones and in *cc2d2a*-/- photoreceptors (A-D)** Quantification for various parameters generated by tracking of tagged Rab8 particles on 10-minute long videos recorded at 1 frame/second. 13 photoreceptors (PRs) per group (5 different conditions) were analyzed: PRs expressing mCherry-Rab8a in wt cones (orange circles), wt rods (orange squares) and *cc2d2a*-/- rods (blue inverted triangles) as well as PRs expressing mCherry- Rab8ba in wt rods (orange triangles) and *cc2d2a*-/- rods (blue diamonds). Each dot in the scatter plots represents the average value of all the particles present in 1 PR. Only particles present in ≥10 frames were analyzed. Bars indicate average and standard deviations. **(A)** Particle displacement measured as the distance between the first set of coordinates and the last set of coordinates. Average displacement was close to 1 μm for all conditions. **(B)** Maximum speed of particles (largest distance traveled between two consecutive time points) was about 1 μm/s in all conditions. **(C)** Particle directionality measured as the ratio of distance spanned in the lateral axis over the distance spanned in the apico-basal axis. A predominantly apico-basal movement was observed in all conditions; Rab8ba particles displayed a more lateral movement in *cc2d2a*-/- compared to wt rods. **(D)** Total trajectory traveled by particles during the entire duration of the recording. Average trajectory was close to 20 μm for all conditions. Given the much more limited displacement, this indicates that the particles shuffle substantially within the inner segment, as visible on the videos. **(E)** Average cross-sectional surface area of tracked particles, calculated from the number of pixels constituting each particle. **(F)** Proportion of puncta coming in proximity with the BB = “contacting the BB”. * p < 0.*01*, ** p < 0.*01*, n.s. not significant, Mann-Whitney U-test. Note consistent results between Rab8a-particles in cones and rods, between Rab8a and Rab8ba particles and between wt and *cc2d2a*-/- for both paralogs for the majority of parameters.

We thus pursued to track the movement of Rab8a or Rab8ba-tagged particles over 10 minutes in wild-type cones and rods (S4–S6 videos) using the tracking software Ilastik (Fig 6A’–6B’). mCherry-tagged Rab8 particles demonstrated a complex movement pattern with “shuffling” along the apico-basal axis predominantly in the inner segment of photoreceptors (S4–S6 videos). Interestingly, we observed no significant difference in displacement (defined as the distance between the first and last coordinates of the recording: on average 890±28 nm; Fig 7A), maximum speed (defined as the largest distance traveled by a particle between two consecutive frames taken at 1 second intervals, on average 1016±98 nm/s; Fig 7B) or trajectory (total distance traveled during the entire duration of the recording, on average 21±12 μm; Fig 7D) between Rab8a and Rab8ba paralogs or between rods and cones. Tracked particles had a cross-sectional area of around 0.3 μm on average (calculated based on the number of constituting pixels, Fig 7E), which is consistent with the size of polylobulated vesicular structures seen on CLEM (arrows in Fig 4A’). Altogether, our data suggest that Rab8a and Rab8ba have a similar behavior in PRs, which could indicate functional redundancy, and that Rab8a-controlled trafficking occurs similarly in rods and cones.

### Rab8-mediated trafficking is not directly affected by loss of Cc2d2a function

To determine whether the vesicle accumulation observed in *cc2d2a*-/- photoreceptors (PRs) is secondary to a defect in Rab8-mediated trafficking, we compared kinetics of mCherry-Rab8a and -Rab8ba particles between wt and *cc2d2a-/-* rods. Indeed, while mCherry-Rab8 is in part mislocalized diffusely to the cytoplasm in mutant PRs, some mCherry-Rab8 particles are retained in mutant PRs. Using the same video duration and acquisition rate paradigm described above, we observed no major differences in movement kinetics in mutant PRs compared to wt including displacement, speed or particle size (S7 and S8 videos; quantification in Fig 7A–7F). The only variable differing significantly in mutant PRs was the directionality of Rab8ba puncta, which was shifted towards an increased lateral movement in *cc2d2a*-/- rods (Fig 7C). Given that cell-shape is grossly maintained in *cc2d2a*-/- PRs, this difference could be explained by increased difficulty of particle movement through accumulated vesicles. However, when quantifying the relative number of particles reaching the periciliary membrane region and approaching the basal body (BB) (Fig 7F), we did not observe a significant difference for either of the Rab8 paralogs in mutant vs wt PRs. The absolute number of Rab8 particles coming in proximity with the BB varied widely between different PRs in both wt and mutants, ranging from 0 to 10 in wt and 0 to 28 in mutants. We conclude that loss of Cc2d2a function does not directly affect Rab8-trafficking.

### Loss of Cc2d2a function results in mislocalization of the t-SNARE SNAP25 at the periciliary membrane

**Fig 8:**
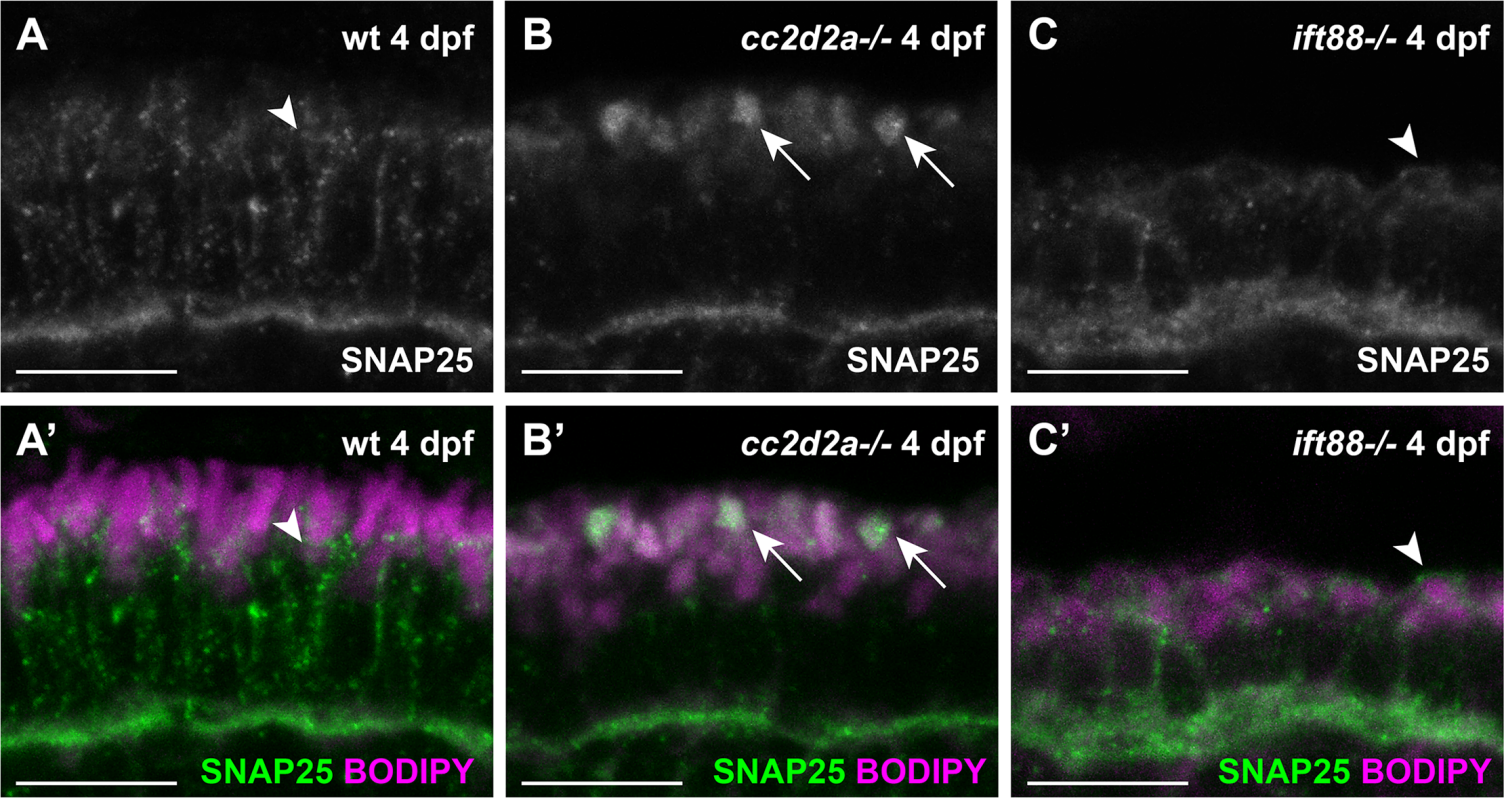
**SNAP25 mislocalizes in *cc2d2a-/-* PRs (A-C’)** 4 dpf cryosections of wild-type (wt) **(A-A’)**, *cc2d2a-/-* **(B-B’)** and *ift88-/-* **(C-C’)** retinae stained for SNAP25 (green in **A’-C’**) and BODIPY (magenta). In wt PRs **(A**-**A’)** SNAP25 localizes along the plasma membrane, between the mitochondrial cluster and the OS (arrowhead) and at the synapse. In *cc2d2a-/-* **(B-B’)**, SNAP25 synaptic localization is preserved but apical mislocalization to a membrane-rich compartment (BODIPY, arrows) is obvious. **(C** and **C’)** Despite absence of OSs in the *ift88-/-* mutant, SNAP25 localizes correctly at the apical membrane of PRs. Scale bars are 10 μm in all panels.

Because the massive accumulation of vesicles in *cc2d2a*-/- PRs occurs only apically and Rab8-trafficking seems unaffected, we hypothesized that the trafficking defect observed in these mutants must happen at late steps of the trafficking process, namely vesicle fusion. Therefore, we focused on the fusion machinery components at the ciliary base. It was previously shown that the t-SNAREs required for the delivery of OCVs in frog and mammalian retina are SNAP25 and Syntaxin3 [25, 40]. We performed immunostaining to determine the localization of these proteins in zebrafish PRs. In wild-type (wt) animals and consistent with previous reports, we found SNAP25 to be localized along the plasma membrane (Fig 8A–8A’), including at the synapse and at the apical membrane between the inner segment (IS) and outer segment (arrowheads in Fig 8A–8A’). In *cc2d2a*-/- PRs, however, SNAP25 was mislocalized apically in a membranous compartment highlighted by BODIPY, while its synaptic localization was unaffected, suggesting again a ciliary-specific effect (Fig 8B–8B’). Moreoever, SNAP25 mislocalization appears to be specific to loss of Cc2d2a, as it was not present in PRs of the ciliary mutant *oval*/*ift88*, defective for intraflagellar transport (IFT) and unable to form OSs, in which SNAP25 signal remains clearly present at the apical membrane just apical to the mitochondrial cluster (Fig 8C–8C’).

**Fig 9:**
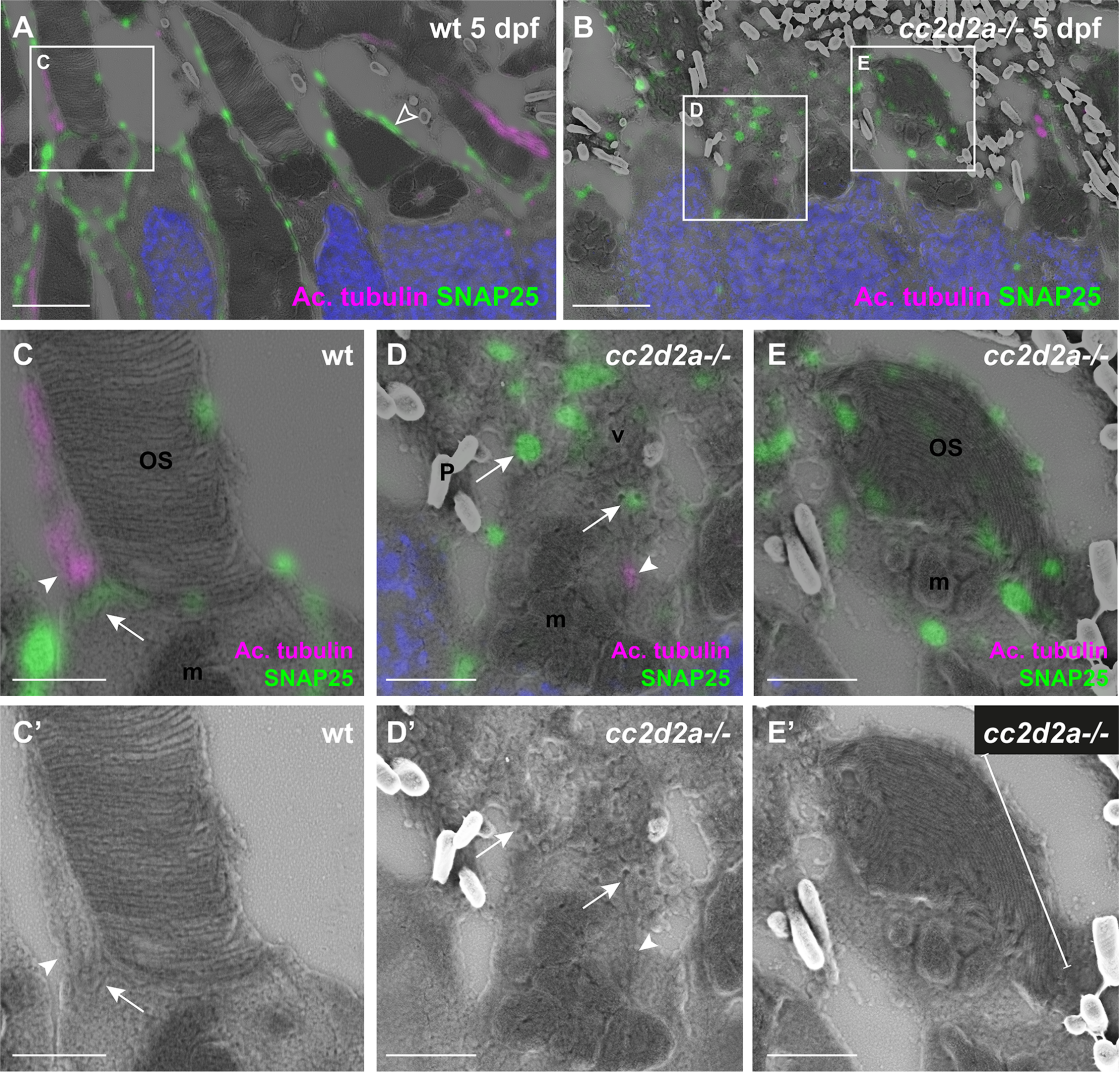
**SNAP25 localizes to wt periciliary membrane and mislocalizes to accumulated vesicles of *cc2d2a-/-* PRs (A-E)** 5 dpf CLEM retinal sections stained for SNAP25 (green), acetylated tubulin (magenta) and DAPI (blue, nuclei). **(C-E)** are higher magnification images of the boxed areas in (A, B) and **(C’-E’)** are the corresponding SEM images. In wt PRs **(A)** SNAP25 is found at the inner segment apical membrane, along the calycal processes (empty arrowhead in A) as well as at the periciliary membrane **(C-C’**, arrow) around the base of the anti-acetylated tubulin-marked cilium **(C-C’**, arrowhead). In contrast, in *cc2d2a-/-* PRs **(B)**, SNAP25 prominently mislocalizes in accumulated vesicles **(D-D’**, arrows) and dysmorphic OSs **(E-E’**, bracket). Arrowheads point to connecting cilia in (C-D’). Scale bars: 4 μm in A-B, 1 μm in C-E’. *OS* outer segment, *m* mitochondria, *N* nuclei, *P* pigment, *v* vesicles, *wt* wild-type.

To determine the precise subcellular localization of SNAP25 in wild-type and mutant PRs, we performed CLEM. In the apical portion of PRs, SNAP25 localized to the apical membrane of the IS, including the periciliary membrane (Fig 9C–9C’, arrow) at the base of the anti-acetylated tubulin-highlighted primary cilium (Fig 9C–9C’, arrowhead), and to the membrane of calycal processes (Fig 9A, empty arrowhead). In *cc2d2a*-/- PRs, however, SNAP25 was mislocalized to accumulated vesicles and to dysmorphic OSs by CLEM (Fig 9D–9D’ and 9E–9E’ respectively). Importantly, this SNAP25 mislocalization was observed as early as 3 dpf (S10 Fig), paralleling the accumulation of vesicles in the apical region of PRs (S11 Fig). Taken together, our data suggest that Cc2d2a is required for the correct localization of SNAP25 at the periciliary membrane from the onset of OS formation.

### Decreased levels of the t-SNAREs Syntaxin3 and SNAP25 and of the Exocyst component Exoc4 in *cc2d2a*-/- retina

**Fig 10:**
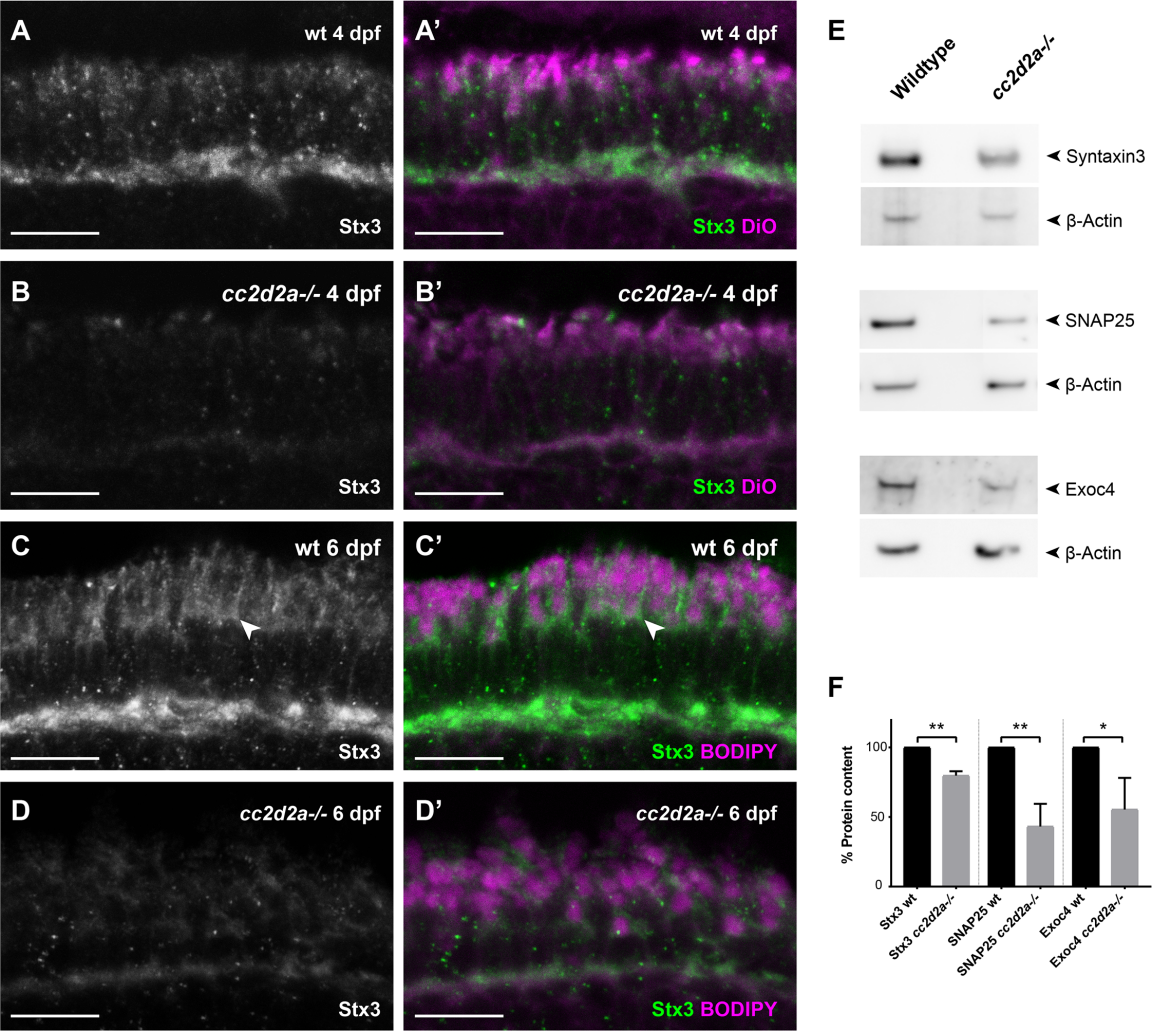
**Proteins involved in OCV fusion are affected by loss of Cc2d2a function** 4 dpf wild-type (wt) **(A-A’)** and *cc2d2a-/-* retinal cryosections **(B**-**B’)** and 6 dpf wt **(C-C’)** and *cc2d2a-/-* retinal cryosections **(D-D’)**, all stained for Syntaxin3 (Stx3) (grayscale in **A-D** and green in **A’-D’**) and counterstained with DiO **(A’,B’)** or BODIPY **(C’,D’)** (both magenta) to label membranes. In wt PRs at both developmental times Stx3 localizes along the plasma membrane (arrowhead), between the mitochondrial cluster and the OS and at the synapse **(A-A’, C-C’)**, similar to SNAP25. While minimal Stx3 mislocalization is visible in *cc2d2a*-/- at 4dpf, a striking decrease in fluorescence intensity is obvious in the mutant **(B, D)**. **(E)** Western blot on whole eye lysates at 6 dpf confirms decreased protein levels of Stx3 in *cc2d2a*-/-. Protein levels of SNAP25 and the Exocyst component Exoc4 are also decreased in *cc2d2a*-/- whole eyes. **(F)** Relative protein content was determined as the ratio of band intensity relative to the housekeeping protein control (beta-actin), averaged for all replicates and repeated in 3 independent blots. Error bars represent standard deviation. *p<0.*05*, ** p< 0.*01*, Student’s t-test. Full western blots are shown in S12 Fig.

We next turned to the second known t-SNARE involved in fusion of OCVs in PRs, Syntaxin3 (Stx3). We found Syntaxin3 localization to mirror SNAP25 localization at the plasma membrane, including the periciliary membrane, in wild-type PRs at 4 dpf and 6 dpf (Fig 10A–10A’ and 10C–10C’, arrowheads). In 4 dpf *cc2d2a*-/- PRs we observed minor mislocalization of Syntaxin3 (Fig 10B–10B’), which was much less prominent than for SNAP25 and decreased further at 6 dpf (Fig 10D–10D’). However, at both time points we consistently observed a strong decrease in fluorescence intensity. We confirmed by western blots that both Syntaxin3 and SNAP25 levels were decreased in whole 6 dpf larval eyes (Fig 10E and 10F and S12 Fig). We next focused our analysis on components of the Exocyst, which is a tethering complex downstream of Rab-mediated targeting and upstream of SNARE-mediated fusion. Specifically, we measured levels of the Sec8 homolog Exoc4, which is a predicted interactor of Rab8, and found these to be decreased in *cc2d2a* mutant eyes (Fig 10E and 10F and S12 Fig). Collectively, our data suggest that loss of Cc2d2a leads to mislocalization and/or depletion of various components required for fusion of OCVs at the periciliary membrane.

## Discussion

Primary cilia are devoted to the transduction of a plethora of signals crucial for embryonic development, adult tissue homeostasis and interpretation of environmental stimuli, such as light [41]. Therefore, regulation of ciliary protein content, in particular of transmembrane receptors and channels, is indispensable for primary cilium function [4]. While the ciliary transition zone (TZ) is thought to act as a gatekeeper in this process [7], its link to upstream protein sorting mechanisms and polarized vesicular trafficking has not been elucidated so far. Our study using CC2D2A as a representative TZ protein indicates that CC2D2A is required for the last steps of trafficking, namely vesicle fusion. Furthermore, our work provides novel evidence in support of a role for Rab8 in opsin-carrier vesicle (OCVs) trafficking in photoreceptors and suggests that this trafficking is only indirectly affected by dysfunction of the TZ.

The vesicle fusion defects observed in *cc2d2a*-/- PRs are secondary to loss of the vesicle tethering and fusion machinery components SNAP25, Syntaxin3 and Exoc4 from the periciliary region. These vesicle fusion defects are ciliary-specific, despite the broader localization of the analyzed SNAREs along the PR cell membrane, since delivery of non-ciliary proteins to other highly organized membrane compartments such as the synapse remains unaffected. Furthermore, SNAP25 mislocalization as seen in *cc2d2a*-/- PRs is not observed in the non-TZ ciliary mutant *ift88-/-*, which also lacks vesicle accumulation [34], strongly suggesting that the fusion machinery defects are specific to loss of Cc2d2a/TZ function.

**Fig 11:**
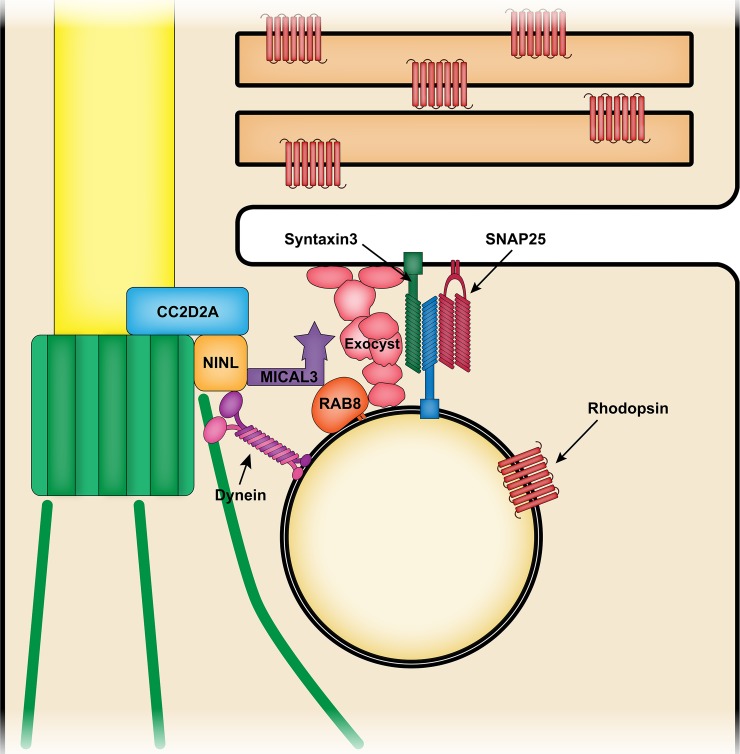
The role of CC2D2A in opsin-carrier-vesicle fusion at the periciliary membrane. Rab8 coats opsin-carrier-vesicles and targets them to the periciliary membrane, where their fusion is mediated by the Exocyst and by SNAREs including SNAP25 and Syntaxin 3 (and by an as yet undefined v-SNARE). CC2D2A at the transition zone is required for correct localization of SNAP25 to the periciliary membrane and provides a docking point for incoming vesicles through its interaction with NINL, which also binds the dynein motor and the Rab8 effector MICAL3. Thus, CC2D2A plays a role in concentrating all components required for correct vesicle fusion at the periciliary membrane.

We have previously shown CC2D2A to interact with NINL [42], a centrosomal protein that also interacts with dynein-dynactin motor proteins and MICAL3, which is a proposed Rab8 effector [43]. Both Rab8 and MICAL3 have catalytic properties that can modify cortical actin cytoskeleton [43–45], thus facilitating vesicle docking [46]. In addition, Rab8 recruits the Exocyst to initiate vesicle fusion [23, 47]. Taken together with the data presented in this work, we propose a model whereby Cc2d2a at the TZ may provide a docking point for incoming OCVs through its interaction with NINL and control the localization of the t-SNAREs required for fusion, thereby bringing all components required for vesicle fusion in proximity with each other at the periciliary region (Fig 11).

The mechanisms underlying SNAP25 mislocalization and vesicle fusion machinery depletion (including SNAP25, Syntaxin3 and Exoc4) in *cc2d2a* mutant PRs remain to be elucidated. Previous work has shown that SNAREs can be downregulated if their interactors are missing or not appropriately localized [48]. Given the observed mislocalization of SNAP25, the cell might try to prevent aberrant fusion of OCVs to the wrong membranes by downregulating Syntaxin3, depleting the Exocyst and reducing the amount of Rab8 engaged on OCVs. SNARE mislocalization was described in the ciliopathy *bbs17-/-* mouse model, where Syntaxin3 and Stx1bp are mislocalized in the OSs of mutant PRs [49]. The authors propose that Bbs17 is required for retrograde transport of proteins out of the ciliary compartment. In our experiments, accumulation of SNAP25-positive vesicles occurred in *cc2d2a*-/- PRs even in the absence of formation of OSs, speaking against a role for Cc2d2a in active retrograde transport.

One possible explanation for SNAP25 mislocalization could be disrupted phospholipid composition of the periciliary membrane. Indeed, recent work determined that the JBTS-protein INPP5E indirectly controls protein content of the ciliary membrane by regulating ciliary phosphoinositides (PIPs) [50]. SNAP25 does not possess a transmembrane domain but is inserted in the target membrane through palmitoylation [51]; thus, its localization might be influenced by the lipid composition of the periciliary membrane. Other proteins regulating intracellular trafficking such as FIP3 or Exocyst subunits contain PIP-binding domains that can be highly specific for certain phospholipids [46, 52]. Moreover, SNAREs are arranged around cholesterol and PIP_2_-rich membrane microdomains [53–55]. OCV delivery was shown to depend on PIP_2_ recognition by FIP3 and to be enhanced by providing docosahexaenoic acid to the PRs [14, 25]. Interestingly, MICAL3 recognizes membrane-associated proteins bound to specific PIPs as the vesicle “landing site” [43, 56]. Given that the ciliary localization of INPP5E depends on the TZ [50, 57], loss of the TZ protein Cc2d2a could impact the balance of phosphoinositides not only in the ciliary, but also in the periciliary membrane, which could in turn lead to mislocalization of SNAP25.

A large body of work has studied OCV trafficking and opsin delivery to the OSs. OCVs are thought to travel from the Golgi to the OS along microtubules using a dynein motor [10, 58]. Multiple players thought to be involved in sorting and targeting of OCVs at various steps along this path include Arf4, ASAP1, Rab11, FIP3 and ultimately Rabin8, which binds and activates Rab8, priming the OCV for its fusion [14]. However, a recent publication has questioned this model, suggesting that Rab8a and Rab8b paralogs are dispensable for rhodopsin transport: in that study, double *rab8a;rab11a* knockout mice and *rab8a* knockout mice with additional expression of dominant negative forms of Rab8b, Rab11a, Rab11b and of the close Rab8-relative Rab10 lack a retinal phenotype [20]. In contrast, our study relying on live imaging of tagged Rab8 and CLEM in photoreceptors supports a role for Rab8 paralogs in rhodopsin transport. We demonstrate presence of Rab8 on vesicular structures containing opsins. Moreover, the movement pattern of Rab8 particles shows apico-basal directionality towards the BB (as expected with movement along microtubules) at a speed consistent with cytoplasmic dynein-driven transport [59] and we show Rab8 to move together with opsin in whole live retina. In addition, our work investigated for the first time both rod and cone PRs with respect to Rab8-trafficking, and found no biologically relevant difference between these PR types. Our findings also support redundancy between Rab8 paralogs, which might extend to other Rabs as well. While one of the Rab8 paralogs might dominate in the physiologic situation, the other Rab8 paralogs, and potentially additional Rabs such as the closest Rab8-relatives Rab10 and Rab13, may be able to take over in case of loss-of-function of Rab8. Rab13 in particular was not investigated in the work by Ying and collaborators [20]. While Rabs are thought to provide membrane specificity, given the large number of Rab genes (close to 70 in humans) and their high degree of sequence conservation, redundancy may be operating in case of disruptions. In this framework, where Rabs act redundantly in trafficking, and SNAREs are distributed along multiple membrane domains, the function of the TZ would be to bring all the components together to the correct place to engage fusion.

Association of mutations in Exocyst components with ciliopathies, in particular JBTS [27, 30], suggest that the mechanisms uncovered in this work to explain the ciliary dysfunction in *cc2d2a-/-* PRs might represent a more general mechanism underlying JBTS. Since about half of JBTS proteins are TZ proteins, abnormal ciliary protein composition through a similar trafficking/fusion defect could represent a common pathogenic mechanism underlying JBTS. Previous evidence for a role of TZ proteins in general, and Cc2d2a in particular, in controlling the protein composition of the cilium was provided by a *Cc2d2a*-knockout mouse model, in which localization of ciliary proteins adenylyl cyclase III, Arl13b, Smoothened and PKD1 was lost from mutant cilia [31]. In a second Cc2d2a mouse knockout, Rab8 signal was lost from the ciliary base, transport vesicles accumulated and ciliogenesis was impaired in *cc2d2a*-/- mice [60]. While the majority of these findings in mice are consistent with our observations in the zebrafish *cc2d2a* mutant model, we do not observe ciliogenesis defects in our *cc2d2a*-/- fish, despite them being true null mutants [33]. Interestingly, only a subset of tissues showed ciliogenesis defects in the first Cc2d2a knockout mouse, suggesting the requirement for Cc2d2a in ciliogenesis to be tissue-specific. Given that vertebrate animal models display very early embryonic lethality in complete absence of cilia, it is unlikely that major ciliogenesis defects would lead to the relatively milder phenotypes of JBTS in humans.

Taken together, our findings suggest that dysfunction of the TZ leads to disorganization of the vesicle fusion machinery, causing accumulation of ciliary-bound vesicles, secondary trafficking defects and aberrant protein content of the ciliary membrane. This is consistent with the ascribed gate-keeper function of the TZ, but suggests a more complex and active role, where the TZ influences both sides of the barrier. Further work will be required to elucidate the precise mechanisms through which individual TZ proteins affect the periciliary membrane, in our quest to uncovering the pathogenesis underlying JBTS.

## Materials and methods

### Ethics statement

All animal protocols were in compliance with internationally recognized and with Swiss legal ethical guidelines for the use of fish in biomedical research and experiments were approved by the local authorities (Veterinäramt Zürich Tierhaltungsnummer 150).

### Zebrafish maintenance and breeding

Zebrafish *(Danio rerio)* were maintained as described [61]. Embryos were raised at 28°C in embryo medium and staged according to development in days post fertilization (dpf) [62]. 0.003% PTU (1-phenyl-2-thiourea) in embryo medium was used to inhibit melanin synthesis during larval development and facilitate fluorescent microscopy.

### Zebrafish lines

The *cc2d2a*^*uw38*^, the *ift88*^*tz288*^ and the *casper* mutants were previously described [32–34, 63]. Stable zebrafish transgenic lines used in this study included *Tg(rhod*:*mCherry-rab8ba) *[33], *Tg(rhod*:*mCherry-rab8a)*, *Tg(tacp*:*mCherry-rab8a)*, *Tg(rhod*:*hCentrin-GFP)*, *Tg(tacp*:*hCentrin-GFP*); the latter four lines were generated in this study (see next paragraph). Constructs injected transiently included *rhod*:*mCherry-rab3aa* (generated in this study) and *hsp70*:*rhod-GFP* (a gift from J. Malicki [37]).

### Construct and transgenic line generation

RT-PCR was performed using cDNA obtained from whole larvae at 5 dpf to generate a Gateway (Invitrogen) p3’ entry clone including the full length coding sequence for Rab3aa (NM_001003419.1). The following primers were used (attB site sequences are written in lowercase):

Rab3aa attB2r: 5’-ggggacagctttcttgtacaaagtggcgATGGCTTCAGCGAATGATGC-3’

Rab3aa attB3: 5’-ggggacaactttgtataataaagttgtTTAGCAAGCGCAGTCCTTGT-3’

Gateway (Invitrogen) recombination was performed using the Tol2 system [64]. p5’ entry clones included the rod-specific rhodopsin promoter (gift from the Link lab) or the cone-specific alpha-transducin promoter (tacp, [65]). mCherry or GFP middle-entry clones and p3’ entry clones containing the human Centrin sequence (gift from the Link lab), the Rab3aa and the Rab8a zebrafish sequence (NM_001089562; gift from the Beales lab) were used to generate N-terminal fusions of Rab proteins or Centrin. The resulting constructs were co-injected with Tol2 transposase mRNA as previously described [66] into 1-cell stage embryos. Injected fish were either imaged (transients) or raised and further generations outcrossed to *casper* zebrafish for at least two generations.

### Annotation of Rab cDNAs

As gene predictions within GenBank are produced by automated processes which may contain errors, Rab cDNA sequences used in this study were manually annotated. Sequences were identified and annotated using combined information from expressed sequence tags and genome databases (GeneBank, http://www.ncbi.nlm.nih.gov; Ensembl, http://www.ensembl.org/index.html). Human and mouse sequences were used as initial query (for more details on sequence annotation see [67]).

### Phylogenetic analysis

The phylogenetic analysis was performed on the Phylogeny.fr platform (http://www.phylogeny.fr/) comprising the following steps [68]. Sequences were aligned using MUSCLE (v3.7) [69] configured for highest accuracy (MUSCLE with default settings). Length of sequences varied between 214 and 700 amino acids. After alignment, ambiguous regions (i.e. containing gaps and/or poorly aligned) were removed using Gblocks (v0.91b) [70]. The following parameters were implemented: The minimum length of a block after gap cleaning was set to 5; positions with a gap in less than 50% of the sequences were selected in the final alignment if they were within an appropriate block; all segments with contiguous non-conserved positions bigger than 8 were rejected; minimum number of sequences for a flank position were 55%. After curation, 203 amino acids were chosen for further analysis. The phylogenetic tree was reconstructed using the maximum likelihood method implemented in the PhyML program (v3.0 aLRT) [71]. The default substitution model was selected assuming an estimated proportion of invariant sites (of 0.000) and 4 gamma-distributed rate categories to account for rate heterogeneity across sites. The gamma shape parameter was estimated directly from the data (gamma = 0.728). Reliability for internal branch was assessed using the aLRT test [72]. Graphical representation and edition of the phylogenetic tree were performed with TreeDyn (v198.3) and the svg file imported into CorelDraw (version x5; Corel Corporation Ottawa, Canada) for final editing.

### Synteny analysis

Synteny analysis was done using the synteny database (http://syntenydb.uoregon.edu/synteny_db/) [73]. Parameters were adjusted to a sliding window size of 50, and several genes in the vicinity of *rab8b* were used for additional syntenic comparison. The final graph was assembled using a combination of the obtained synteny files and adjusted in size and color using CorelDraw. Several synteny hits in the output files were used as initial queries for a tBLASTx search against the zebrafish or human database (ncbi nr/nt database) to verify orthology.

### Transmission Electron Microscopy (TEM)

Zebrafish larvae were fixed overnight at 4°C in a freshly prepared mixture of 2.5% glutaraldehyde and 2% formaldehyde (FA) in 0.1M sodium cacodylate buffer (pH 7.4). After rinsing in buffer, specimens were washed in 1% osmiumtetroxide and 1% potassiumferrocyanide in 0.1 M sodiumcacodylate buffer (pH 7.4), during 2 h at room temperature. After rinsing, tissues were dehydrated through a graded series of ethanol and embedded in epon. Ultrathin sections (70nm) comprising zebrafish eyes were collected on formvar coated grids, subsequently stained with 2% uranyl acetate and Reynold’s lead citrate, and examined with a transmission electron microscope Philips CM-100.

### Correlative light and electron microscopy (CLEM)

A detailed protocol is available at JoVE [35]. Briefly, 5 dpf old larvae were euthanised in Tricaine (ethyl 3-aminobenzoate methanesulfonate, Sigma-Aldrich) and their sectioned heads fixed in 4% formaldehyde/0.025% glutaraldehyde in cacodylate buffer overnight at 4°C. Eyes were dissected out in fixative, washed in PBS, embedded in 12% gelatin in 0.1 M PBS at 40°C, cooled down and immersed and stored in 2.3 M sucrose at 4°C. Prepared samples were frozen in liquid nitrogen and sectioned with a cryo-ultramicrotome (Ultracut EM FC6, Leica Microsystems). 100 nm ultrathin sections were transferred to a 7 × 7 mm silicon wafer (Si-Mat Silicon Materials) and stored at 4°C.

Sections were stained with mouse anti-rhodopsin (4D2, gift from R. Molday, University of British Columbia) 1:100, rabbit anti-SNAP25 1:250 (StressGen Biotechnologies, VAP-SV002), chicken anti-mCherry 1:50 (Abcam), mouse anti-acetylated tubulin 1:100 (Sigma Aldrich, clone 6-11B-1) and counterstained when necessary with BODIPY TR methyl ester (Invitrogen) 1:100 and DAPI (4′,6-diamidino-2-phenylindole dihydrochloride) 1:100. Alexa Fluor 488-conjugated secondary antibodies (Life Technologies) were used at 1:200. After confocal laser scanning microscopy, samples were postfixed with 0.1% glutaraldehyde in PBS, covered with a thin layer of methylcellulose and coated with 10 nm Platinum/Carbon by rotary shadowing at an angle of 8 degrees. SEM images were taken using a Zeiss Supra 50 VP and a Zeiss Auriga 40 SEM. Alignment of light and electron microscopy images was done with the open-source platform Fiji, based on manually inserted landmarks from the nuclear DAPI signal and using the TrakEM2 plugin. At least 3 animals per condition were used for CLEM.

### Live imaging

Transiently injected *Tg(rhod*:*mCherry-rab3aa)* and *Tg(hsp70*:*rhod-GFP)* and stable *Tg(rhod*:*mCherry-rab8a;rhod*:*GFP-hCentrin)*, *Tg(rhod*:*mCherry-rab8ba;rhod*:*GFP-hCentrin)* and *Tg(tacp*:*mCherry-rab8a)* 5 dpf zebrafish larvae were anesthetized in Tricaine, embedded in 1.8% low melting agarose (Lonza), mounted on glass-bottom Petri dishes and covered with Tricaine-supplemented embryo medium. Time-lapse videos from a single focal plane were obtained with a spinning disk microscope using a 60x 1.4 NA objective (Visitron, CSU-W1). The acquisition lasted for 10 min at a rate of 1 frame per second including both channels (excitation lasers at 488 nm and 561 nm). 13 photoreceptors from 4–7 animals per condition were analyzed. All videos shown are displayed with a 15-fold acceleration (15 frames/second) and edited using Fiji and Adobe Premiere Pro CC.

### Tracking and video analysis

Time-lapse live imaging videos were registered using the MultiStackReg plugin in Fiji. To avoid sampling error carryover, only three to four photoreceptors (PRs) were selected per animal for analysis. The analyzed PRs were randomly picked (random.org) from the pool of eligible PRs in the videos. The chosen PRs were first isolated using Fiji followed by signal segmentation and automated tracking using the respective pipelines in the open-source software Ilastik (versions 1.1.5, 1.1.7 and 1.2.0 [74]). Segmentation cues were provided every 5 to 10 frames. Segmented videos were processed for tracking using sigma values of 0.1 for Gaussian blurring and a threshold between 0.29 and 0.31. Potential objects were only considered when they had a minimum size of 2 pixels (1 pixel = 210 nm). Optimized tracking parameters were: max. number of objects per merger = 1, division weight = 10, transition weight = 3, appearance cost = 9, disappearance cost = 47. Objects were not considered divisible.

The selected software outputs were the raw coordinates of the particle barycenter in each frame (used to calculate the maximum speed and the displacement (measured as the distance between the first and the last set of coordinates of a particle)). Directionality was expressed as the quotient of the maximum distance spanned in the lateral X axis over the maximum distance spanned in the apico-basal Y axis (after correcting the coordinates in the reference Cartesian system using Euler angle rotations: x_new_ = x_original_ * cosθ + y_original_ * sinθ; and y_new_ = y_original_ * cosθ—x_original_ * sinθ). Thus, results <1 represent apico-basally directed movement. Particle size was calculated based on the number of consituting pixels and the surface of individual pixels (pixel size 210nm x 210nm) and expressed as the cross-sectional surface area (surface of each pixel = 0.044 μm^2^, 10 pixels = 0.44 μm^2^). A particle approaching the BB was considered to reach the periciliary membrane (determined as a “contact”) when the barycenter coordinates of the particle were within a 3 pixel radius (630nm) from the barycenter of the BB.

### Immunofluorescence

Zebrafish larvae were fixed in 4% PFA at room temperature (RT) for 30 min, embedded in Neg50 (Richard-Allan Scientific) and cryosectioned following standard protocols. Non-specific binding was blocked using PBDT (PBS, 1% DMSO, 0.5% Triton X-100, 2mg/ml BSA) with 10% goat serum for 30 minutes at RT before incubation with primary antibodies overnight at 4°C. Primary antibodies were rabbit anti-Syntaxin3 (1:400, Alomone labs, ANR-005), rabbit anti-SNAP25 (1:1000, StressGen Biotechnologies, VAP-SV002), mouse monoclonal anti-Rab8a (1:100, Novus Biologicals, clone 3G1), rabbit anti-Cacna1fa (1:5000, a gift from Michael Taylor, University of Wisconsin [75]), rabbit anti-blue opsin (1:250, gift from David Hyde) and rabbit anti green opsin (1:400, gift from David Hyde, University of Notre Dame). Secondary antibodies were Alexa Fluor-conjugated goat anti-rabbit or goat anti-mouse IgG (1:400, Life Technologies). BODIPY TR methyl ester (1:300, Invitrogen) or Vybrant DiO (1:200, ThermoFischer) was applied for 20 min and nuclei were counterstained with DAPI. Confocal laser scanning microscopy was performed on a Leica HCS LSI or a Leica SP5 microscope. All immunofluorescence experiments were performed at least in duplicate with at least 10 animals per condition.

### Western blot

6 dpf zebrafish larvae were anesthetized in Tricaine. Whole larval eyes were dissected and collected in PBS and transferred to urea buffer (65 mM Tris HCl pH 6.75, 8 M urea, 20% glycerine, 5% SDS, 5% β-mercaptoethanol) containing protease inhibitors (cOmplete EDTA-free Protease Inhibitor Cocktail, Roche). Samples were subsequently lysed using a Sonopuls HD 2070 sonicator, and separated on Mini-PROTEAN TGX 4–15% precast polyacrylamide gels (Bio-Rad). Proteins were transferred to a polyvinylidene difluoride (PVDF) membrane (Invitrogen). Nonspecific antibody binding was inhibited by incubation in PBST (PBS, 0.05% Tween-20) supplemented with 3% skimmed milk powder. Membranes were probed using antibodies against Syntaxin3 (rabbit, 1:2000, Alomone labs, ANR-005), rSec8 (mouse, 1:1000, Enzo, clone 14G1), SNAP25 (rabbit, 1:5000, StressGen Biotechnologies, VAP-SV002), Rab8 (mouse, 1:500, Novus Biologicals, clone 3G1) and β-actin (mouse, 1:1000, Sigma, A1978). HRP-conjugated goat anti-mouse (1:3000, Merck) or anti-rabbit (1:5000, Merck) were used to detect proteins of interest and subsequently visualized by chemiluminescence using luminol/peroxide substrate (SuperSignal West Dura Extended Duration Substrate, Life Technologies) and an ImageQuant LAS 4000 imager.

### Statistics

All statistical tests were run using GraphPad Prism. Student’s t-tests were used for pairwise comparisons between wild-type (wt) and *cc2d2a-/-* to analyze tracking and western blot results. Western blot results were first normalized to the wt levels. Because parameters relative to periciliary membrane contact do not have a normal distribution, we used a Mann-Whitney U-test instead of a t-test. ANOVA was used to compare kinetic parameters between wt Rab8a cones, wt Rab8a rods and wt Rab8ba rods.

## Supporting information

S1 Video Rab8 co-movement with rhodopsin. Live imaging of heat-shock induced **rhodopsin-GFP** expression (green in merge) and mCherry-**Rab8a** (magenta in merge) in cone photoreceptors (PRs). Video acquired at a rate of 1 frame/s and displayed at 15 frames/s. Arrowheads point to two co-localizing GFP-positive and mCherry-positive puncta that are moving together. OS outer segments, IS inner segments, S synapse.
(MOV)

S2 Video Centrin GFP Rab8a Cherry. Live imaging of rhodopsin promoter-driven expression of **GFP-Centrin** (green) and mCherry-**Rab8a** (magenta) in **rod** photoreceptors (PRs). Video acquired at a rate of 1 frame/s and displayed at 15 frames/s. A line marks the synapse and an arrow points to a GFP-positive basal body (BB) at the beginning of the video. Note the shuffling apico-basal movement of the mCherry-positive puncta. A second arrow points to a PR where a punctum approaches the BB and disappears.
(MOV)

S3 Video Rods Rab3aa WT. Live imaging of rhodopsin promoter-driven expression of mCherry-**Rab3aa** in **rod** photoreceptors (PRs). Video acquired at a rate of 1 frame/s and displayed at 15 frames/s. A dashed line marks the outline of a rod PR with the synapse facing the bottom. Local movement can be observed mainly at the synapse, where endogenous Rab3 is predicted to localize and no significant shuffling movement is observed in the inner segment. OS outer segments, IS inner segments, S synapse.
(MOV)

S4 Video Rods Rab8a WT. Live imaging of rhodopsin promoter-driven expression of mCherry-**Rab8a** in **rod** photoreceptors (PRs). Video acquired at a rate of 1 frame/s and displayed at 15 frames/s. Note the shuffling apico-basal movement of the mCherry-positive particles. OS outer segments, IS inner segments, S synapse.
(MOV)

S5 Video Cones Rab8a WT. Live imaging of transducin promoter-driven expression of mCherry-**Rab8a** in **cone** photoreceptors. Video acquired at a rate of 1 frame/s and displayed at 15 frames/s. Note the shuffling apico-basal movement of the mCherry-positive particles. OS outer segments, IS inner segments, S synapse.
(MOV)

S6 Video Rods Rab8ba WT. Live imaging of rhodopsin promoter-driven expression of mCherry-**Rab8ba** in **rod** photoreceptors. Video acquired at a rate of 1 frame/s and displayed at 15 frames/s. Note the shuffling apico-basal movement of the mCherry-positive particles. OS outer segments, IS inner segments, S synapse.
(MOV)

S7 Video Rods Rab8a MUT. Live imaging of rhodopsin promoter-driven expression of mCherry-**Rab8a** in **rod** photoreceptors (PRs) of a ***cc2d2a-/-*** animal. Video acquired at a rate of 1 frame/s and displayed at 15 frames/s. Note the absence of puncta in most of the expressing PRs but the normal movement of the persisting puncta compared to wild-type animals. IS inner segments, S synapse.
(MOV)

S8 Video Rods Rab8ba MUT. Live imaging of rhodopsin promoter-driven expression of mCherry-**Rab8ba** in **rod** photoreceptors (PRs) of a ***cc2d2a-/-*** animal. Video acquired at a rate of 1 frame/s and displayed at 15 frames/s. Note the absence of puncta in most of the expressing PRs. An arrow points to a particle moving more in the lateral axis, which coexists with another particle displaying clear apico-basal movement in the same PR. IS inner segments, S synapse.
(MOV)

S1 Fig *cc2d2a*-/- retinae do not undergo degeneration at early developmental stages. (A-B”) TUNEL assay on 4 dpf retinal cryosections of wild-type (A-A”) and *cc2d2a* mutants (B-B”). Note the limited number of TUNEL positive cells (B’ and green in B) in mutant retinae. Membranes are counterstained with BODIPY (red in A-B) and nuclei with DAPI (A”-B” and blue in A-B). Also note the normal organisation of mutant retina, including the photoreceptor (PR) cell layer, visible with DAPI in (B”) compared to wild-type in (A”). (C-E) Higher magnification views of the PR cell layer in wild-type (C), *cc2d2a* mutant (D) and *ift88* mutant (E) 4 dpf larvae. Note the normal nuclear morphology in *cc2d2a*-/- PRs compared to wild-type and compared to the degenerating *ift88* retina which displays rounded nuclei (arrows in E), gaps (arrowheads in E) and a globally thinned PR cell layer (bracket in E). (F) Quantification of TUNEL positive cells in wild-type (green inverted triangles) and in *cc2d2a* mutant (red triangles) at 3 and 4 dpf. While the amount of cell death is statistically significantly increased at 3 dpf in mutant compared to wild-type, it remains minimal (on average 4.6% of evaluated nuclei are TUNEL positive in mutants, compared to 0.8% in wild-type). At 4 dpf, no increase in cell death is observed in *cc2d2a* mutant retinae compared to wild-type. NS non significant, *** p<0.001, t-test, n>20 animals for each condition. Quantification was performed on confocal stacks of identical dimensions in wild-type and mutant.
(PDF)

S2 Fig BB docking occurs normally in *cc2d2a*-/- PRs. 5 dpf CLEM sections of transgenic *tg(tacp*:*GFP-hCentrin)* wild-type (wt) **(A)** and *cc2d2a*-/- **(B)** fish expressing GFP-tagged centrin and counterstained with DAPI (blue, nuclei). GFP-Centrin-labeled basal bodies (BBs) (green) localize at the apical membrane of both wt and mutant animals. **(A’)** BB is docked right below the outer segment (white arrowhead), apical to the daughter centriole (yellow arrowhead) in wt. **(B’)** BB (white arrowhead) is localized correctly in *cc2d2a*-/- PRs even when the OSs appear dysmorphic and disorganized. Scale bars: 4 μm in A-B and 2 μm in A’-B’. *OS* outer segment, *m* mitochondria, *N* nucleus, *wt* wild-type.
(PDF)

S3 Fig Accumulated vesicles in *cc2d2a*-/- PRs at 3 dpf contain opsin. **(A**) 3 dpf correlative light and electron microscopy (CLEM) image of a *cc2d2a*-/- retina stained with 4D2 (green) to label rhodopsin and red-green cone opsin and with DAPI (blue, nuclei). Arrows point to mislocalized opsin inside the cell body. **(B-C)** Higher magnification images of the boxed regions in **(A)**. **(B)** Accumulated apical vesicles contain opsin in 4D2-positive PRs. **(B’)** corresponding scanning electron microscopy image only of **(B)**. **(C)** Some stacking of membranes in OSs can be occasionally observed above accumulating vesicles. Scale bars: 4 μm in **A** and 1 μm in **B-C**. *OS* outer segment, *m* mitochondria, *N* nucleus, *v* vesicular structures.
(PDF)

S4 Fig Polarized trafficking of transmembrane proteins not targeted to cilium occurs normally in *cc2d2a*-/- PRs. Retinal cryosections of 4 dpf **(A-B)** and 6 dpf **(C-D)** wild-type (wt) **(A, C)** and *cc2d2a*-/- **(B, D**) zebrafish, stained with an antibody against Cacna1fa (green), an L-type calcium channel that localizes to the synapse, and counterstained with DAPI (nuclei, blue) and BODIPY (outer segments and mitochondrial cluster, magenta). **(A’-D’)** Close-ups of the synaptic regions boxed in A-D. Cacna1fa (grey) in the mutant **(B’** and **D’)** presents a similar localization at synapses as in wt **(A’** and **C’)** at 4 and 6 dpf. Scale bars: 10 μm in all figures.
(PDF)

S5 Fig Synteny of the zebrafish *rab8b* genes. Genes flanking the rab8b orthologs located on zebrafish chromosomes 7 and 25 and human chromosome 15 are shown. Rab8b genes are highlighted in red. Note that the current NCBI number XM_021470743.1 for Rab8ba represents a currently mis-assembled fusion of rab8ba and megf8-UTR sequences. The localization within the chromosome is given in the scale bar. Note that for better overview parts of the zebrafish chromosome 25 are not drawn to scale. Orthologous genes between human (blue) and corresponding genes on zebrafish chromosomes 7 (green) and 25 (yellow), are depicted. The black lines linking corresponding genes indicate the relative position of the genes on the chromosome and point out the single zebrafish ortholog. Human genes with two corresponding zebrafish orthologs are highlighted by dark red lines. Note that some genes in the region of zebrafish chromosome 25, highlighted by a red box, have their corresponding orthologs on a region of human chromosome 15 that is 30Mb away from the human RAB8B and that the downstream region of the zebrafish rab8ba gene has its corresponding orthologs (shown in purple) on human chromosome 16. *Dre* Danio rerio, *Hsa* Homo sapiens.
(PDF)

S6 Fig The zebrafish possess two *Rab8b* paralogs. Rab sequences of the species indicated were used for phylogenetic reconstructions. Ancient fish species (lacking the teleost specific whole genome duplication) are shown in dark red, teleost species are shown in bright red, the amphibian species *Xenopus tropicalis* is shown in green, Sauropsidia are marked in orange and mammals are depicted in blue. As an outgroup to root the tree, *Rab15* sequences from the three major species were included. Note that all teleosts have two *Rab8b-like* genes. The *Rab8b* paralog used in our studies (*Rab8ba*) is highlighted by a red arrow.
(PDF)

S7 Fig Homology between Rab8 proteins. Amino acid sequences of the following species were aligned using CLC Main Workbench program (version 6), configured for high accuracy: Human *(Homo sapiens)* hsa, mouse *(Mus musculus)* mmu, zebrafish *(Danio rerio)* dre, torafugu *(Takifugu rubripes)* tru, medaka *(Oryzias latipes)* ola. Conservation is displayed as a bar graph (pink boxes) and the sequence logo (occurring amino acids at a given position and their relative abundance are indicated by letter size). Note that variations in the first 110 amino acids are very rare and are only observed in very few of the included sequences.
(PDF)

S8 Fig Comparison of transgenic Rab8 expression with endogenous Rab8 localization. An anti-Rab8 antibody **(B, E** and green in **C, F)** recognizes mCherry-tagged Rab8a (**A,** magenta in **C**) and mCherry-tagged Rab8ba **(D**, magenta in **F)** on retinal cryosections of 5 dpf zebrafish (arrows). The same antibody **(H** and magenta in **I)** recognizes endogenous Rab8 on non-transgenic tissue in a punctated pattern that localizes to the inner segment where it co-localizes with endogenous green opsin **(G** and green in **I,** arrows). Anti-Rab8 antibody signal is also very prominent over the retinal pigment epithelium (RPE) but not inside the DiO labelled OSs (cyan in I). Additional puncta are also visible at the synapse and between the nuclei. Such basal punctate localization pattern is partly consistent with mCherry localisation in the transgenic lines which is occasionally seen in such basal regions of the PRs (see time-lapse videos as well). Given that the transgenic lines analyzed express mCherry-Rab8 only in PRs, the relevance of the anti-Rab8 RPE signal cannot be evaluated. **(J)** A western blot of wild-type whole eyes at 5 dpf probed with the same anti-Rab8 antibody revealed a strong band with the expected size for Rab8 (23.57 KDa). In addition, two other bands were visible on western blot (also acknowledged by the manufacturer: https://www.novusbio.com/products/rab8a-antibody-3g1_h00004218-m02), indicating that the antibody may recognize additional epitopes. Thus, in the absence of Rab8a/Rab8ba/Rab8bb triple knockout mutants, it is not possible to determine the specificity of the anti-Rab8 RPE staining and of the additional puncta with certainty. Scale bars: 10 μm in all panels.
(PDF)

S9 Fig Retinae of rhod:mCherry-rab8a are structurally healthy. **(A**) Transmission electron microscopy image of a wild-type 5dpf *tg(rhod*:*mCherry-rab8a)* retina. The retinal structure remains normal and extension of outer segments is unaffected. Variable accumulation of membrane-bound structures in inner segment regions is observed in a subset of PRs as a consequence of the overexpression of the transgenic construct (absent in **A’,** present in **A”**). Scale bars: 10 μm in A and 2 μm in **A’** and **A”**. *OS* outer segment, *m* mitochondria, *N* nucleus, *v* vesiculo-tubular structures.
(PDF)

S10 Fig SNAP25 mislocalizes in OSs and accumulated vesicles of *cc2d2a-/-* PRs at 3 dpf. **(A**) 3 dpf correlative light and electron microscopy (CLEM) image of a *cc2d2a*-/- retina stained with anti-SNAP25 (green) and DAPI (blue, nuclei). **(B-C’)** Higher magnification images of the boxed regions in **(A)**. SNAP25 mislocalizes in misshapen outer segments **(B)** and accumulated vesicles **(C)**. **(B’** and **C’)** are SEM images only of (B and C). Arrows point to vesicular structures where SNAP25 mislocalizes. Scale bars: 4 μm in A and 1 μm in B-C’. *OS* outer segment, *m* mitochondria, *N* nucleus, *P* pigment, *v* vesicular structures.
(PDF)

S11 Fig Comparison of *cc2d2a* and *ift88* mutant retinae indicates that vesicle accumulation in PRs is not a general non-specific defect secondary to any ciliary dysfunction. **(A-C)** Transmission electron microscopy images of 3 dpf wild-type **(A)**, *cc2d2a* mutant **(B)** and *ift88* mutant **(C)** retinae. Note the accumulation of vesicular structures and abnormal membrane stacks in *cc2d2a* mutants, while no vesicles are found in the inner segments of *ift88* mutants.
(PDF)

S12 Fig Complete western blots for Syntaxin3, Exoc4 and SNAP25. **(A** and **A’)** Lysate triplicates of wt and *cc2d2a*-/- whole eyes probed for **(A)** Syntaxin3 and **(A’)** b-actin in the same blot. b-actin appears at later exposure times **(A’). (B** and **B’)** Lysate duplicates of wt and *cc2d2a*-/- whole eyes probed for **(B)** Exoc4 and **(B’)** b-actin after stripping. **(C** and **C’)** Lysate triplicates of wt and *cc2d2a*-/- whole eyes probed for **(C)** SNAP25 and **(C’)** b-actin in the same blot. b-actin appears at earlier exposure times **(C’).**
(PDF)

S1 Table Live imaging analysis spreadsheets. Data used to generate the plots in Fig 7. The tab “Kinetics” contains the data for the various kinetic parameters analyzed (Displacement, Trajectory, Size and Maximum Speed). The column headings indicate the condition (“wt Rab8a cones” for example). Each line gives the average result for all the puncta analyzed in a given photoreceptor. The tab “Contacts” indicates the proportion and the absolute number of particles coming in proximity to the BB (“contacting”); each line represents the result for one photoreceptor.
(XLSX)
