# Supplementary figures and images for: Loss-of-function of the ciliopathy protein Cc2d2a disorganizes the vesicle fusion machinery at the periciliary membrane and indirectly affects Rab8-trafficking in zebrafish photoreceptors
Source: PLoS Genet. 2017 Dec 27;13(12):e1007150. doi: 10.1371/journal.pgen.1007150 (PMC5760100; doi:10.1371/journal.pgen.1007150)

S1 Fig. *cc2d2a*<sup>-/-</sup> retinæ do not undergo degeneration at early developmental stages

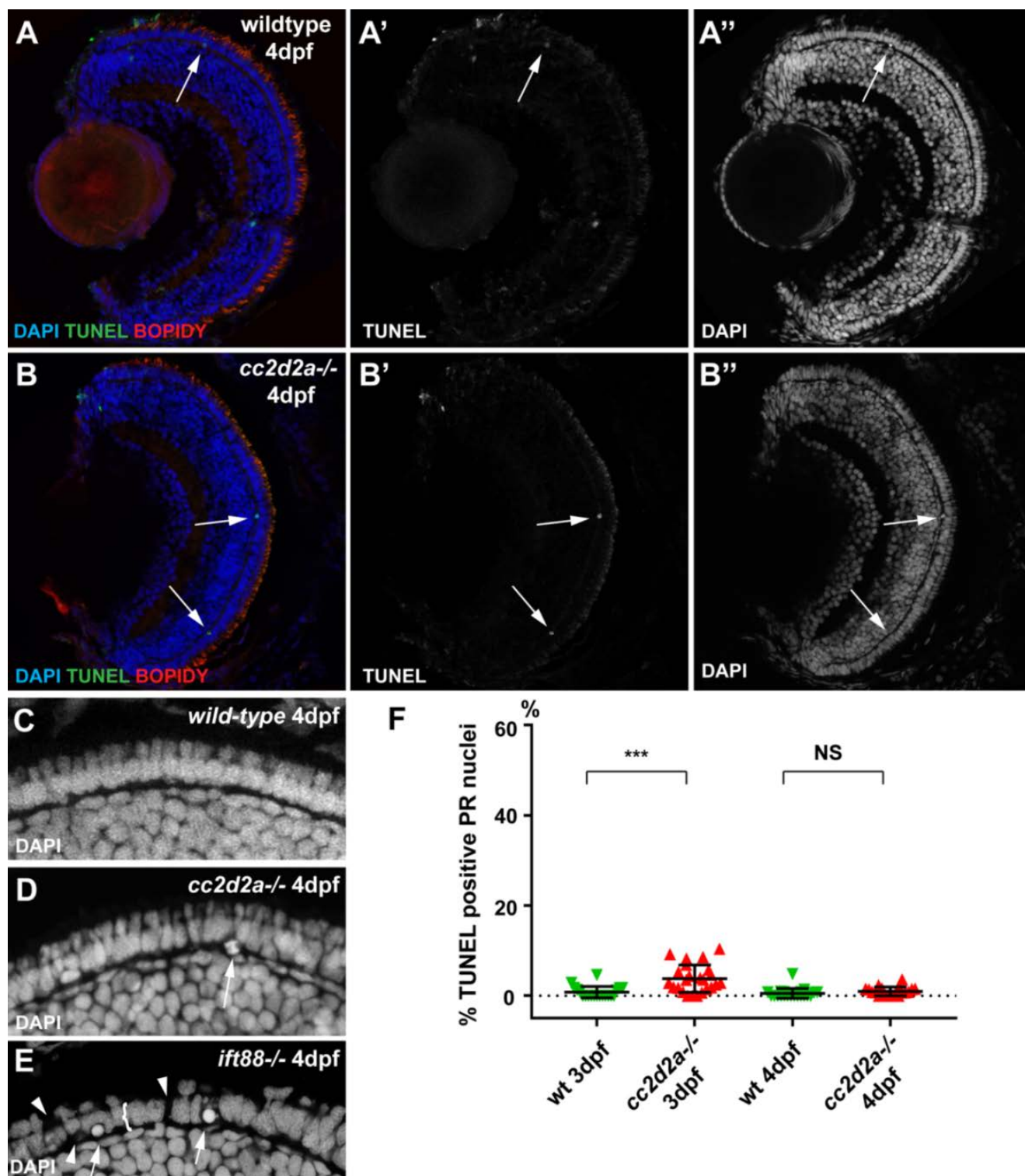

Supplement: S1 Fig — (A-B”) TUNEL assay on 4 dpf retinal cryosections of wild-type (A-A”) and cc2d2a mutants (B-B”). Note the limited number of TUNEL positive cells (B’ and green in B) in mutant retinae. Membranes are counterstained with BODIPY (red in A-B) and nuclei with DAPI (A”-B” and blue in A-B). Also note the normal organisation of mutant retina, including the photoreceptor (PR) cell layer, visible with DAPI in (B”) compared to wild-type in (A”). (C-E) Higher magnification views of the PR cell layer in wild-type (C), cc2d2a mutant (D) and ift88 mutant (E) 4 dpf larvae. Note the normal nuclear morphology in cc2d2a-/- PRs compared to wild-type and compared to the degenerating ift88 retina which displays rounded nuclei (arrows in E), gaps (arrowheads in E) and a globally thinned PR cell layer (bracket in E). (F) Quantification of TUNEL positive cells in wild-type (green inverted triangles) and in cc2d2a mutant (red triangles) at 3 and 4 dpf. While the amount of cell death is statistically significantly increased at 3 dpf in mutant compared to wild-type, it remains minimal (on average 4.6% of evaluated nuclei are TUNEL positive in mutants, compared to 0.8% in wild-type). At 4 dpf, no increase in cell death is observed in cc2d2a mutant retinae compared to wild-type. NS non significant, *** p<0.001, t-test, n>20 animals for each condition. Quantification was performed on confocal stacks of identical dimensions in wild-type and mutant. (PDF) [file pgen.1007150.s009.pdf]

S2 Fig. BB docking occurs normally in *cc2d2a*<sup>-/-</sup> PRs

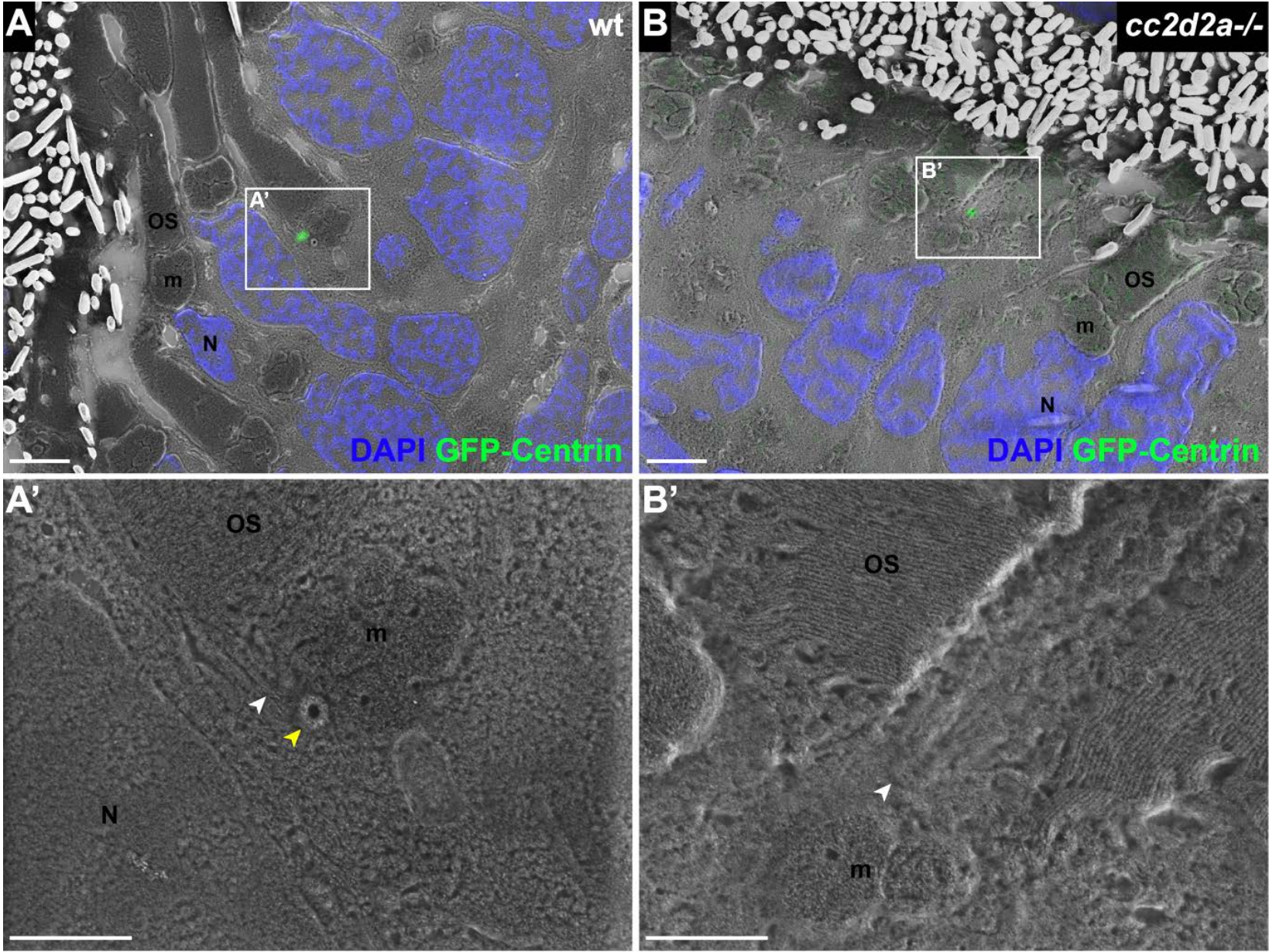

Supplement: S2 Fig — 5 dpf CLEM sections of transgenic tg(tacp:GFP-hCentrin) wild-type (wt) (A) and cc2d2a-/- (B) fish expressing GFP-tagged centrin and counterstained with DAPI (blue, nuclei). GFP-Centrin-labeled basal bodies (BBs) (green) localize at the apical membrane of both wt and mutant animals. (A’) BB is docked right below the outer segment (white arrowhead), apical to the daughter centriole (yellow arrowhead) in wt. (B’) BB (white arrowhead) is localized correctly in cc2d2a-/- PRs even when the OSs appear dysmorphic and disorganized. Scale bars: 4 μm in A-B and 2 μm in A’-B’. OS outer segment, m mitochondria, N nucleus, wt wild-type. (PDF) [file pgen.1007150.s010.pdf]

S3 Fig. Accumulated vesicles in *cc2d2a*<sup>-/-</sup> PRs at 3 dpf contain opsin

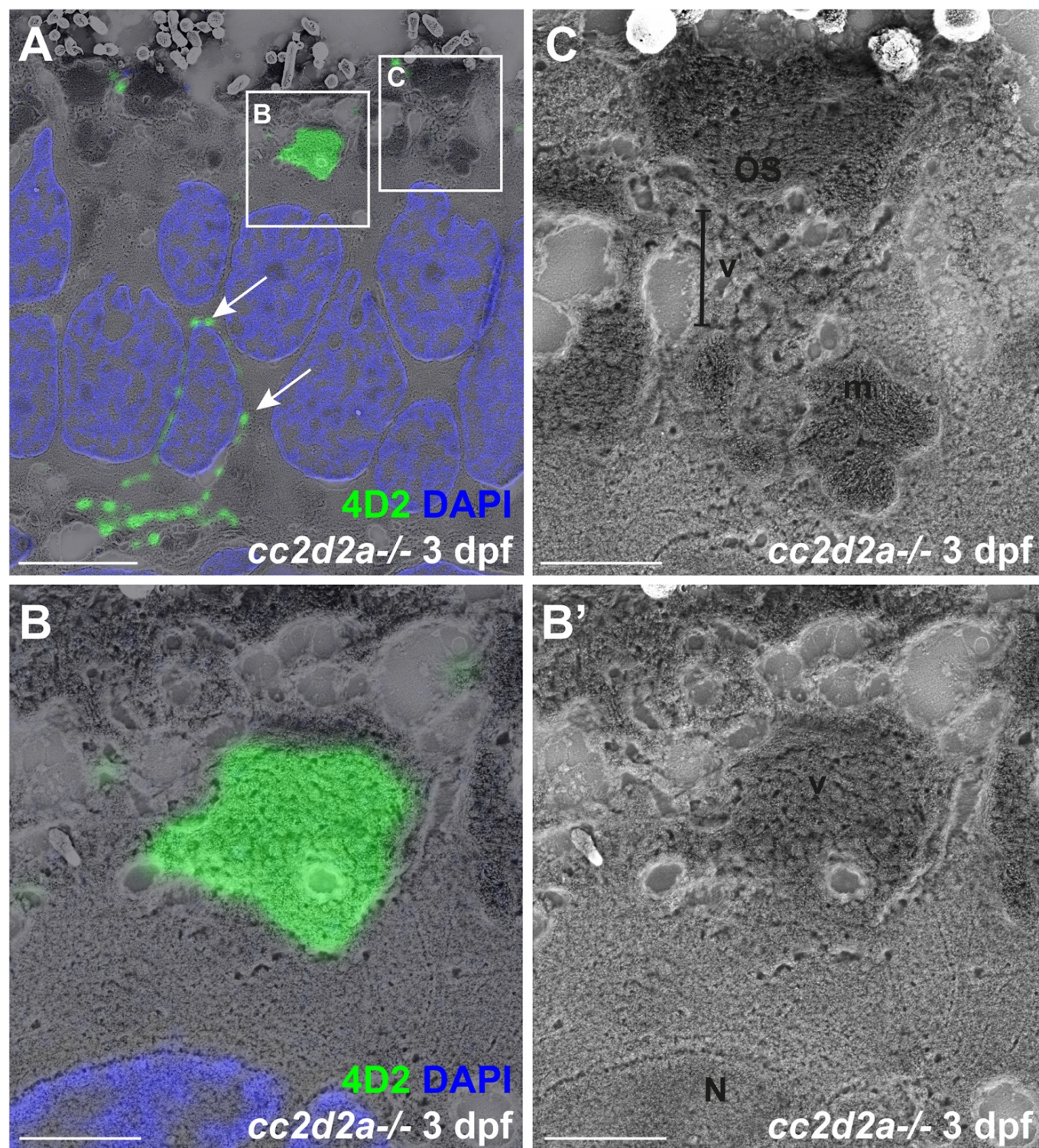

Supplement: S3 Fig — (A) 3 dpf correlative light and electron microscopy (CLEM) image of a cc2d2a-/- retina stained with 4D2 (green) to label rhodopsin and red-green cone opsin and with DAPI (blue, nuclei). Arrows point to mislocalized opsin inside the cell body. (B-C) Higher magnification images of the boxed regions in (A). (B) Accumulated apical vesicles contain opsin in 4D2-positive PRs. (B’) corresponding scanning electron microscopy image only of (B). (C) Some stacking of membranes in OSs can be occasionally observed above accumulating vesicles. Scale bars: 4 μm in A and 1 μm in B-C. OS outer segment, m mitochondria, N nucleus, v vesicular structures. (PDF) [file pgen.1007150.s011.pdf]

S5 Fig. Synteny of the zebrafish *rab8b* genes

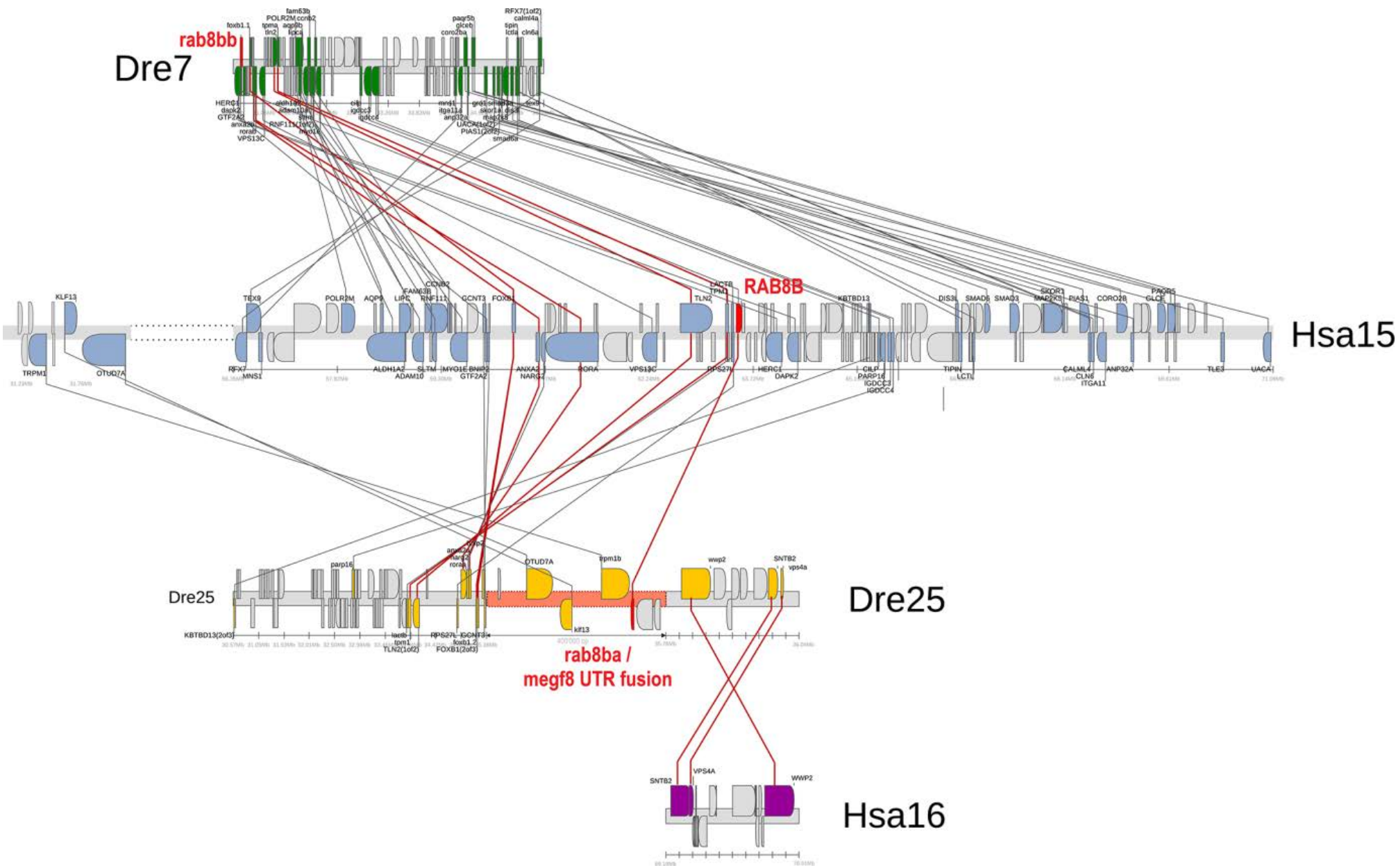

Supplement: S5 Fig — Genes flanking the rab8b orthologs located on zebrafish chromosomes 7 and 25 and human chromosome 15 are shown. Rab8b genes are highlighted in red. Note that the current NCBI number XM_021470743.1 for Rab8ba represents a currently mis-assembled fusion of rab8ba and megf8-UTR sequences. The localization within the chromosome is given in the scale bar. Note that for better overview parts of the zebrafish chromosome 25 are not drawn to scale. Orthologous genes between human (blue) and corresponding genes on zebrafish chromosomes 7 (green) and 25 (yellow), are depicted. The black lines linking corresponding genes indicate the relative position of the genes on the chromosome and point out the single zebrafish ortholog. Human genes with two corresponding zebrafish orthologs are highlighted by dark red lines. Note that some genes in the region of zebrafish chromosome 25, highlighted by a red box, have their corresponding orthologs on a region of human chromosome 15 that is 30Mb away from the human RAB8B and that the downstream region of the zebrafish rab8ba gene has its corresponding orthologs (shown in purple) on human chromosome 16. Dre Danio rerio, Hsa Homo sapiens. (PDF) [file pgen.1007150.s013.pdf]

S6 Fig. The zebrafish possess two *Rab8b* paralogs

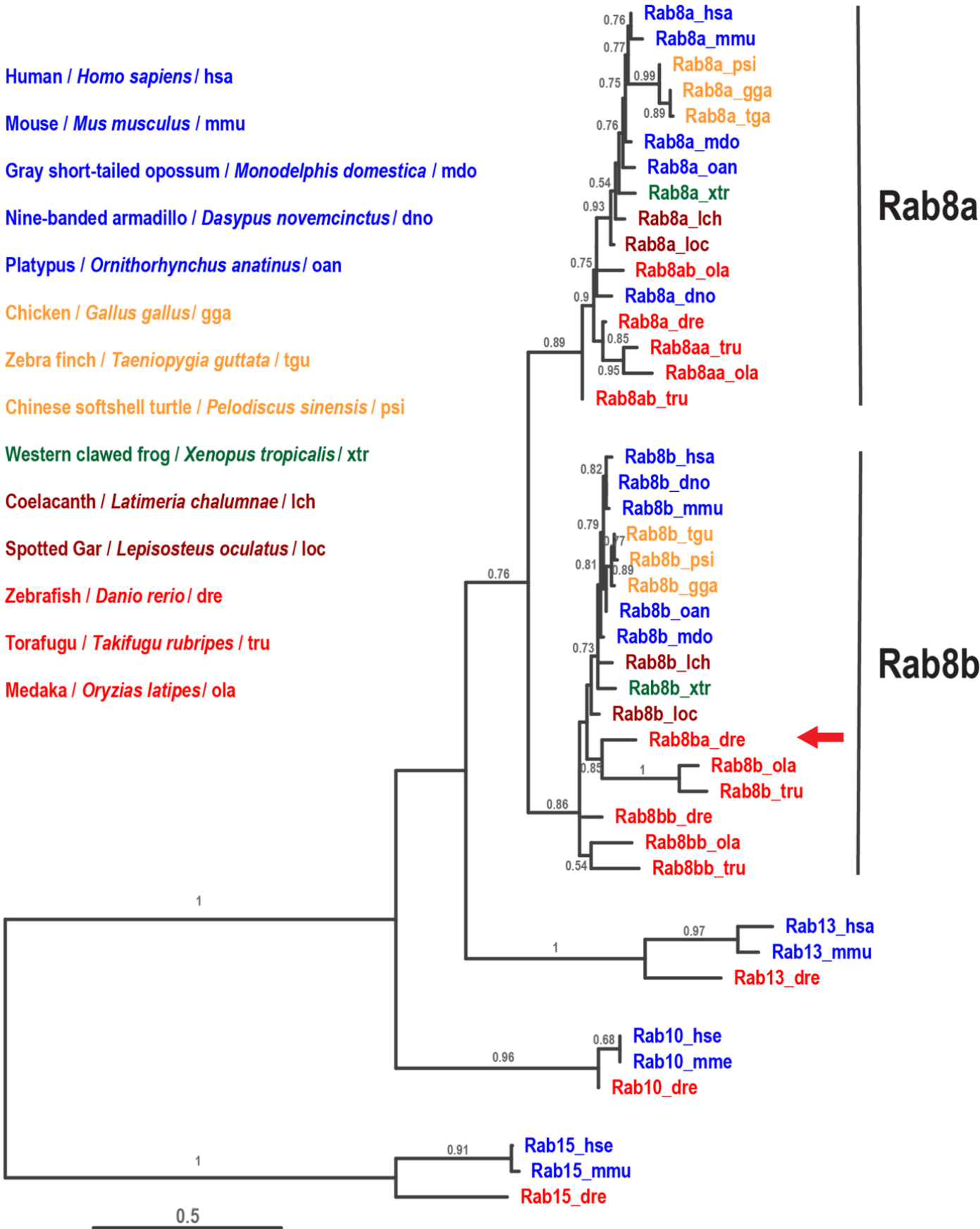

Supplement: S6 Fig — Rab sequences of the species indicated were used for phylogenetic reconstructions. Ancient fish species (lacking the teleost specific whole genome duplication) are shown in dark red, teleost species are shown in bright red, the amphibian species Xenopus tropicalis is shown in green, Sauropsidia are marked in orange and mammals are depicted in blue. As an outgroup to root the tree, Rab15 sequences from the three major species were included. Note that all teleosts have two Rab8b-like genes. The Rab8b paralog used in our studies (Rab8ba) is highlighted by a red arrow. (PDF) [file pgen.1007150.s014.pdf]

S7 Fig. Homology between Rab8 proteins

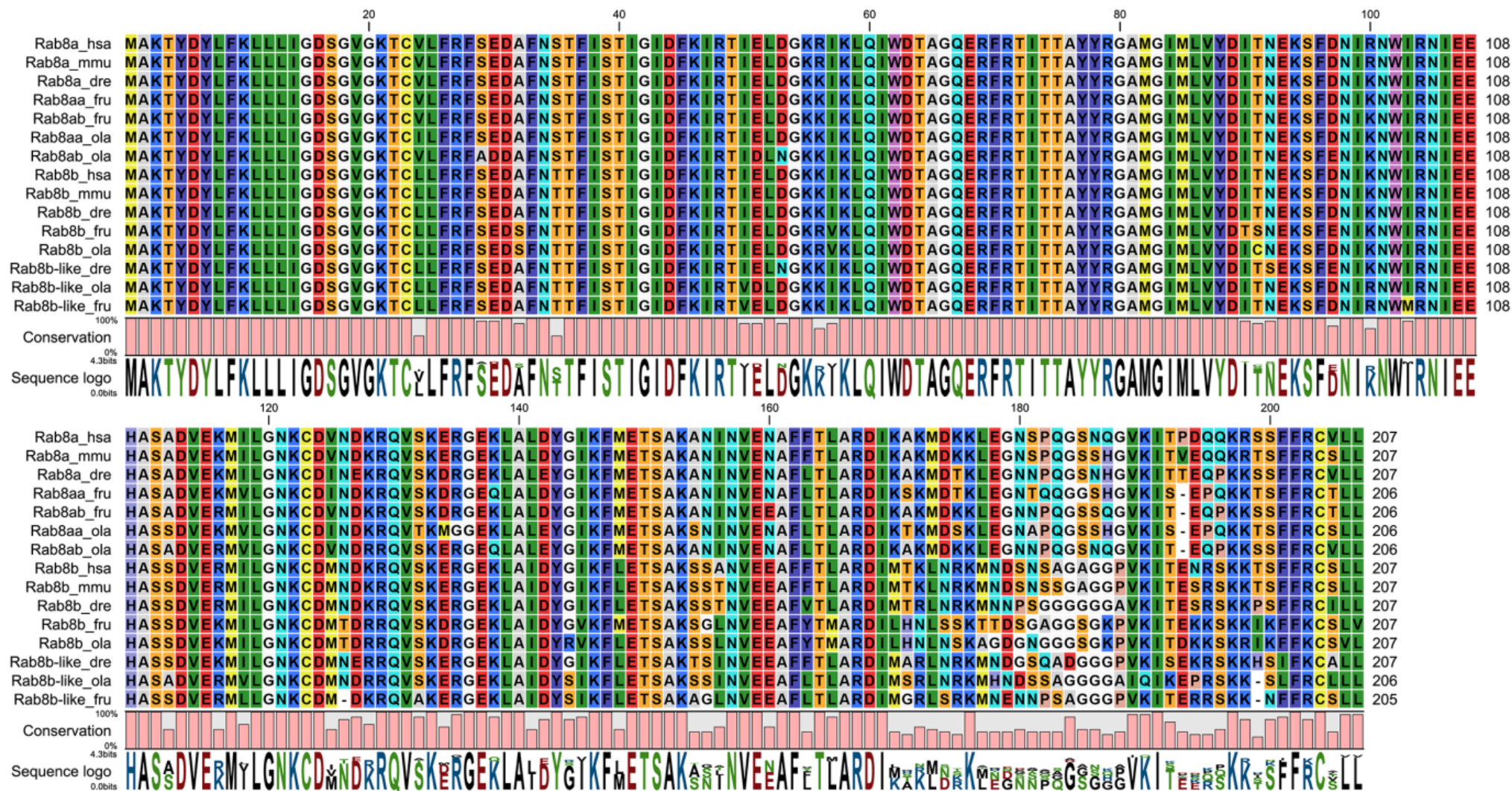

Supplement: S7 Fig — Amino acid sequences of the following species were aligned using CLC Main Workbench program (version 6), configured for high accuracy: Human (Homo sapiens) hsa, mouse (Mus musculus) mmu, zebrafish (Danio rerio) dre, torafugu (Takifugu rubripes) tru, medaka (Oryzias latipes) ola. Conservation is displayed as a bar graph (pink boxes) and the sequence logo (occurring amino acids at a given position and their relative abundance are indicated by letter size). Note that variations in the first 110 amino acids are very rare and are only observed in very few of the included sequences. (PDF) [file pgen.1007150.s015.pdf]

S8 Fig. Comparison of transgenic Rab8 expression with endogenous Rab8 localization

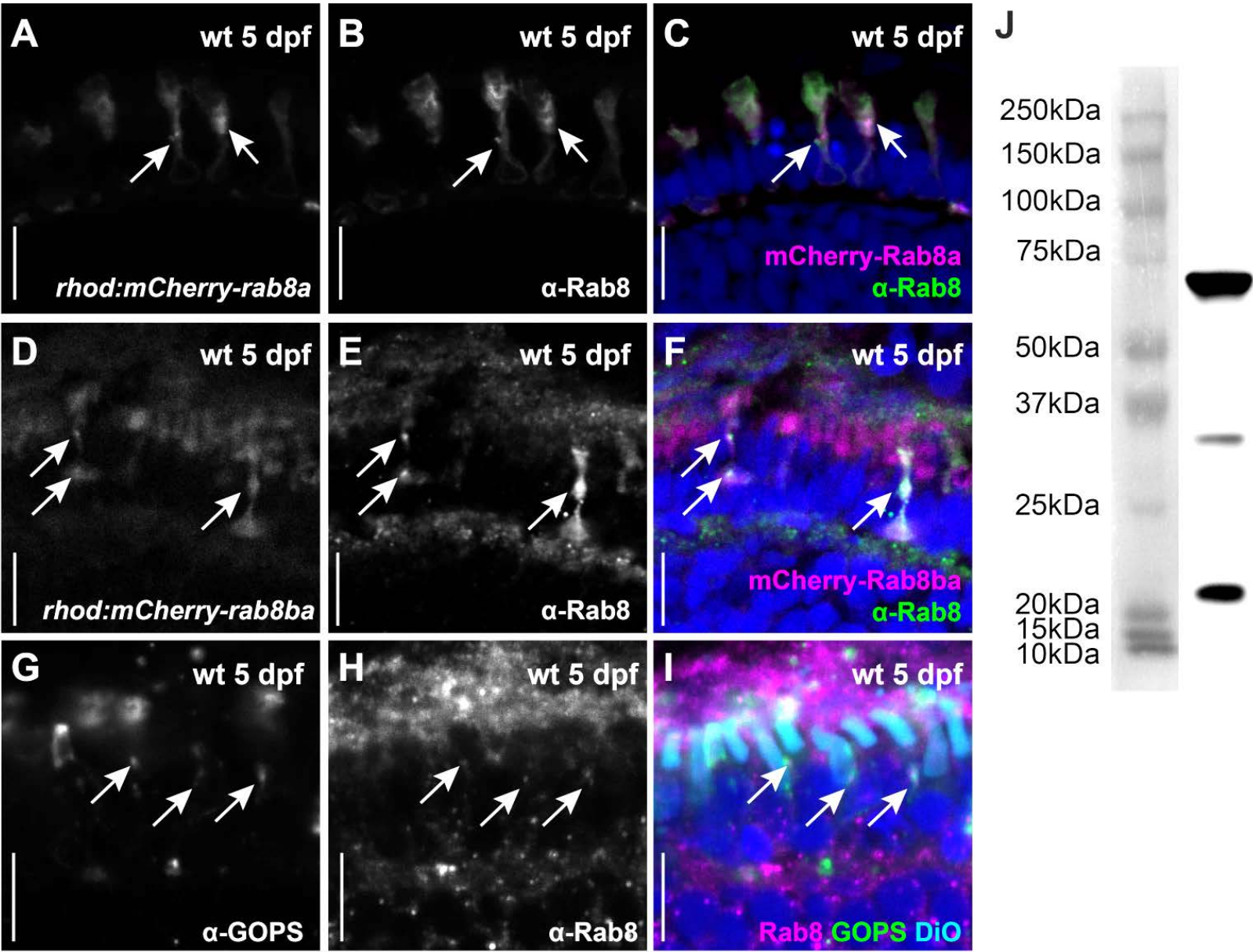

Supplement: S8 Fig — An anti-Rab8 antibody (B, E and green in C, F) recognizes mCherry-tagged Rab8a (A, magenta in C) and mCherry-tagged Rab8ba (D, magenta in F) on retinal cryosections of 5 dpf zebrafish (arrows). The same antibody (H and magenta in I) recognizes endogenous Rab8 on non-transgenic tissue in a punctated pattern that localizes to the inner segment where it co-localizes with endogenous green opsin (G and green in I, arrows). Anti-Rab8 antibody signal is also very prominent over the retinal pigment epithelium (RPE) but not inside the DiO labelled OSs (cyan in I). Additional puncta are also visible at the synapse and between the nuclei. Such basal punctate localization pattern is partly consistent with mCherry localisation in the transgenic lines which is occasionally seen in such basal regions of the PRs (see time-lapse videos as well). Given that the transgenic lines analyzed express mCherry-Rab8 only in PRs, the relevance of the anti-Rab8 RPE signal cannot be evaluated. (J) A western blot of wild-type whole eyes at 5 dpf probed with the same anti-Rab8 antibody revealed a strong band with the expected size for Rab8 (23.57 KDa). In addition, two other bands were visible on western blot (also acknowledged by the manufacturer: https://www.novusbio.com/products/rab8a-antibody-3g1_h00004218-m02), indicating that the antibody may recognize additional epitopes. Thus, in the absence of Rab8a/Rab8ba/Rab8bb triple knockout mutants, it is not possible to determine the specificity of the anti-Rab8 RPE staining and of the additional puncta with certainty. Scale bars: 10 μm in all panels. (PDF) [file pgen.1007150.s016.pdf]

S9 Fig. Retinae of rhod:mCherry-rab8a are structurally healthy

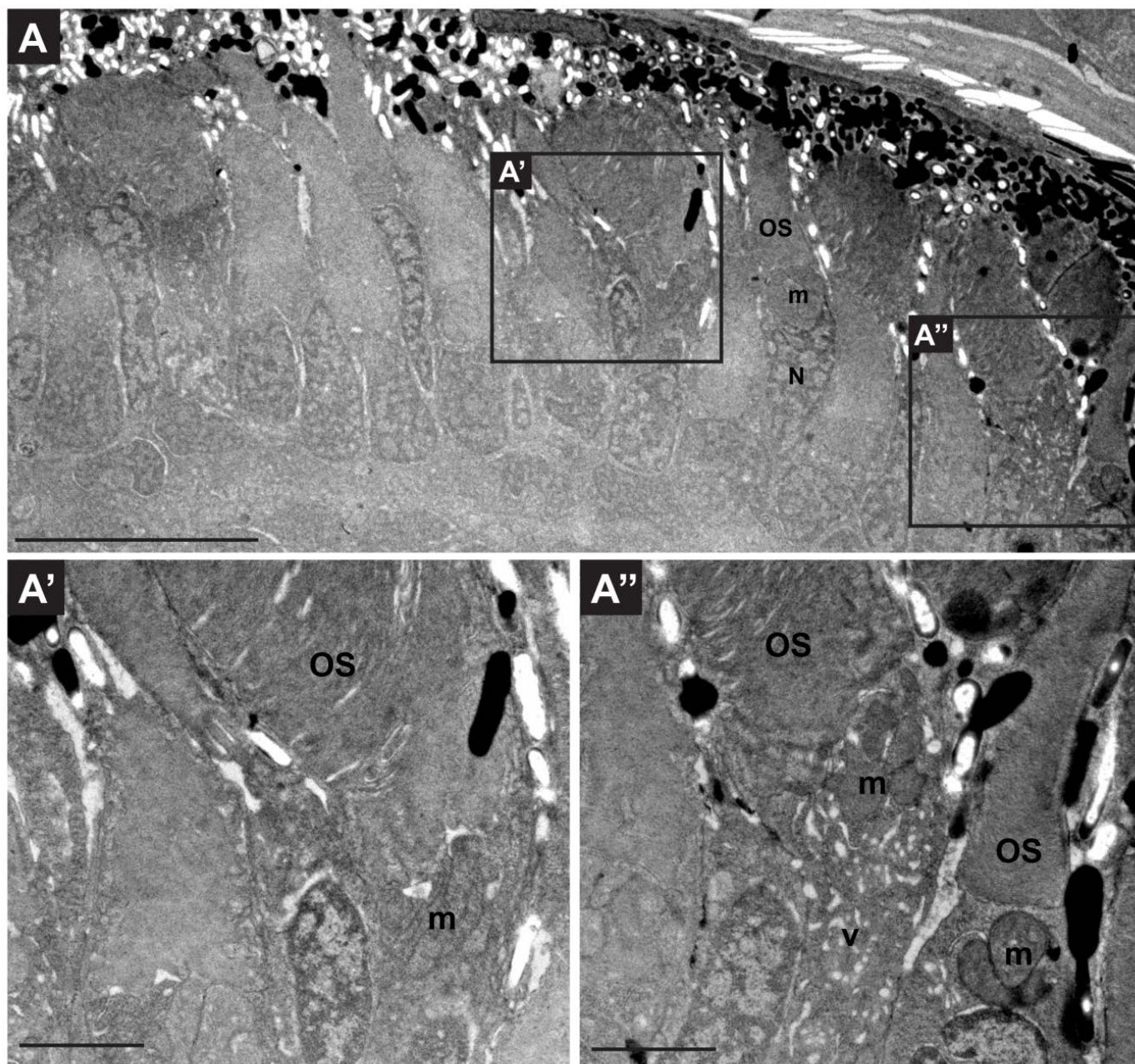

Supplement: S9 Fig — (A) Transmission electron microscopy image of a wild-type 5dpf tg(rhod:mCherry-rab8a) retina. The retinal structure remains normal and extension of outer segments is unaffected. Variable accumulation of membrane-bound structures in inner segment regions is observed in a subset of PRs as a consequence of the overexpression of the transgenic construct (absent in A’, present in A”). Scale bars: 10 μm in A and 2 μm in A’ and A”. OS outer segment, m mitochondria, N nucleus, v vesiculo-tubular structures. (PDF) [file pgen.1007150.s017.pdf]

S10 Fig. SNAP25 mislocalizes in OSs and accumulated vesicles of *cc2d2a*<sup>-/-</sup> PRs at 3 dpf

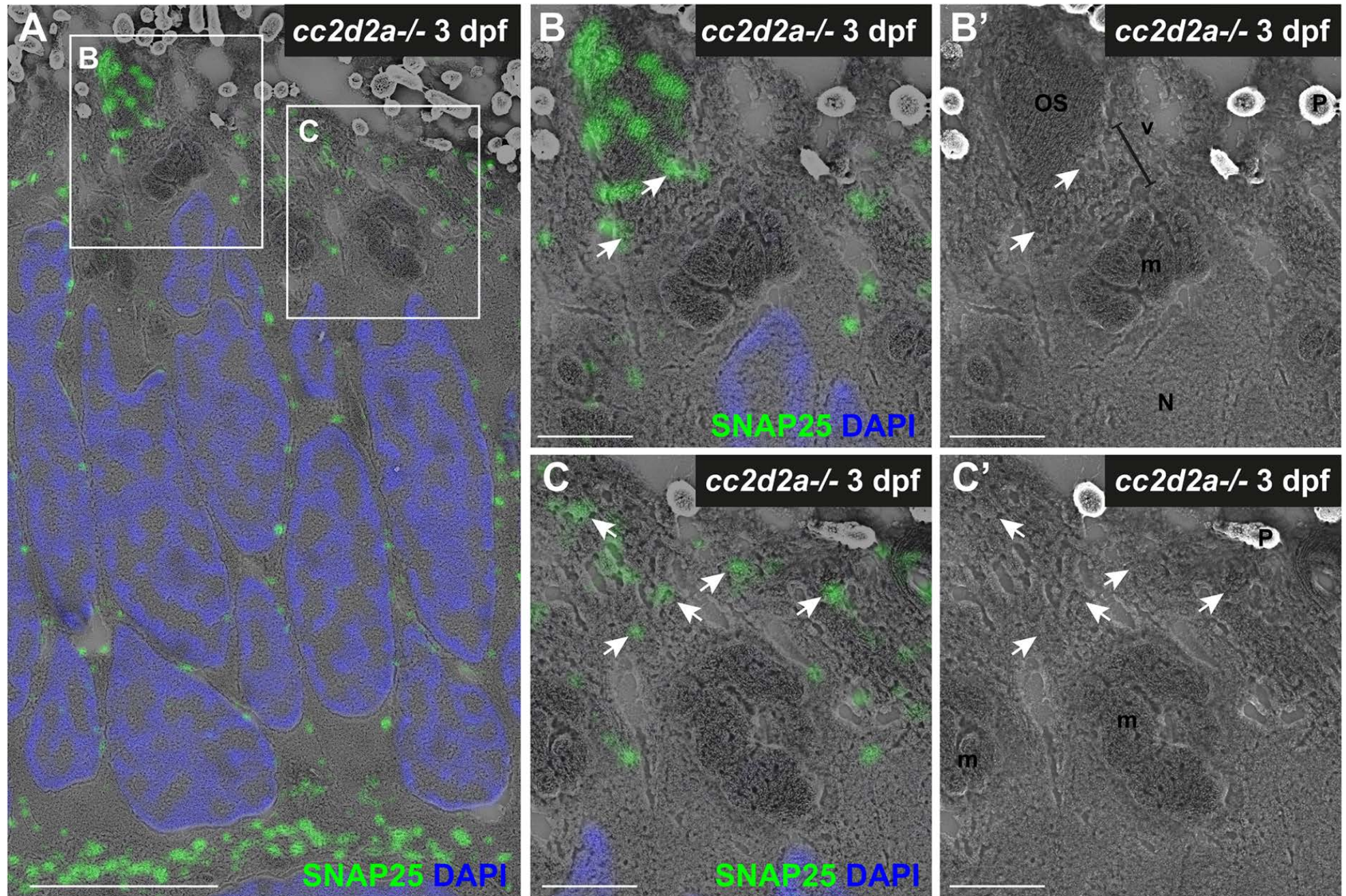

Supplement: S10 Fig — (A) 3 dpf correlative light and electron microscopy (CLEM) image of a cc2d2a-/- retina stained with anti-SNAP25 (green) and DAPI (blue, nuclei). (B-C’) Higher magnification images of the boxed regions in (A). SNAP25 mislocalizes in misshapen outer segments (B) and accumulated vesicles (C). (B’ and C’) are SEM images only of (B and C). Arrows point to vesicular structures where SNAP25 mislocalizes. Scale bars: 4 μm in A and 1 μm in B-C’. OS outer segment, m mitochondria, N nucleus, P pigment, v vesicular structures. (PDF) [file pgen.1007150.s018.pdf]

**S12 Fig. Complete western blots for Syntaxin3, Exoc4 and SNAP25**

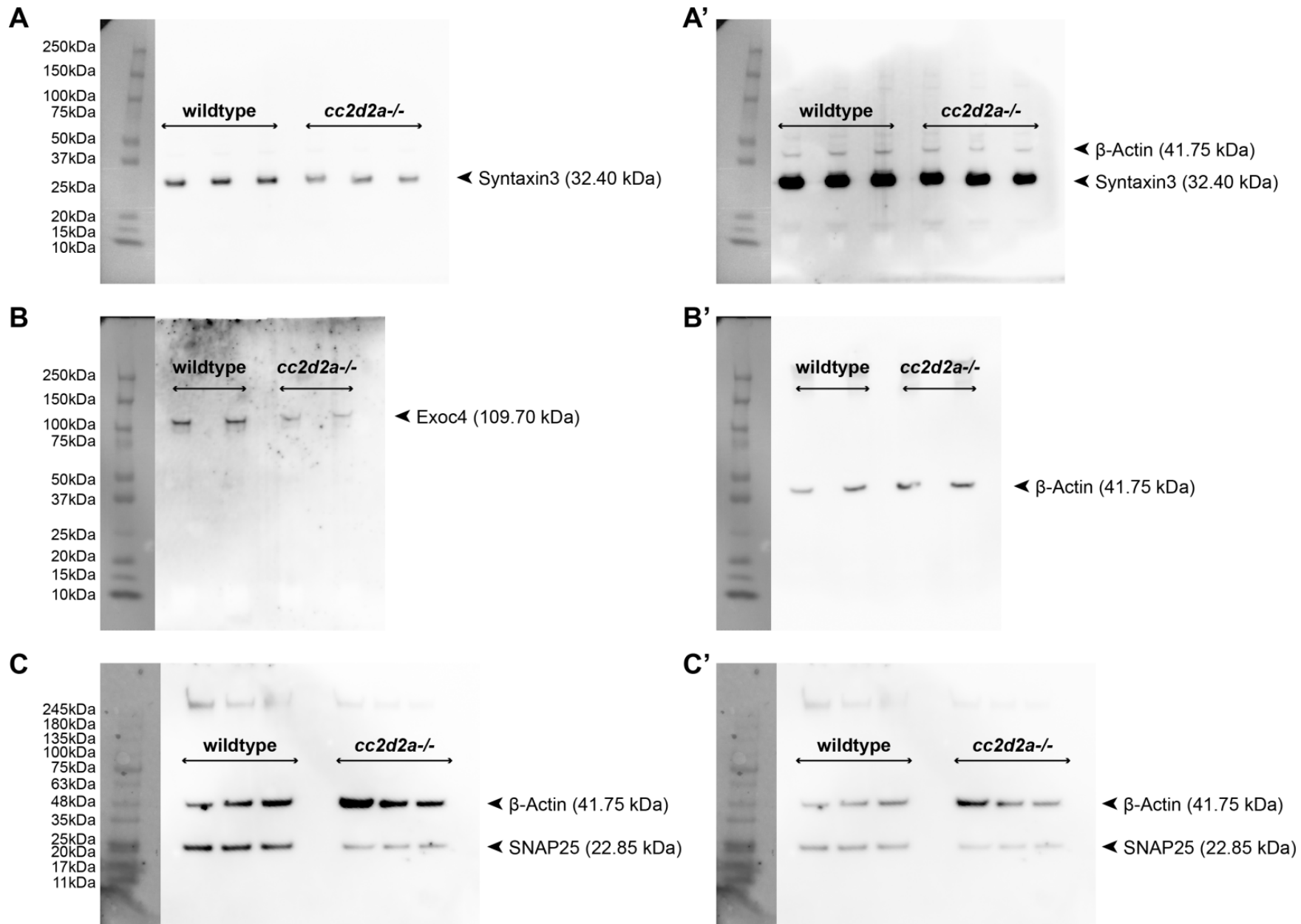

Supplement: S12 Fig — (A and A’) Lysate triplicates of wt and cc2d2a-/- whole eyes probed for (A) Syntaxin3 and (A’) b-actin in the same blot. b-actin appears at later exposure times (A’). (B and B’) Lysate duplicates of wt and cc2d2a-/- whole eyes probed for (B) Exoc4 and (B’) b-actin after stripping. (C and C’) Lysate triplicates of wt and cc2d2a-/- whole eyes probed for (C) SNAP25 and (C’) b-actin in the same blot. b-actin appears at earlier exposure times (C’). (PDF) [file pgen.1007150.s020.pdf]
