# Supplementary material for: Loss-of-function of the ciliopathy protein Cc2d2a disorganizes the vesicle fusion machinery at the periciliary membrane and indirectly affects Rab8-trafficking in zebrafish photoreceptors
Source: PLoS Genet. 2017 Dec 27;13(12):e1007150. doi: 10.1371/journal.pgen.1007150 (PMC5760100; doi:10.1371/journal.pgen.1007150)

S4 Fig. Polarized trafficking of transmembrane proteins not targeted to cilium occurs normally in *cc2d2a-/-* PRs

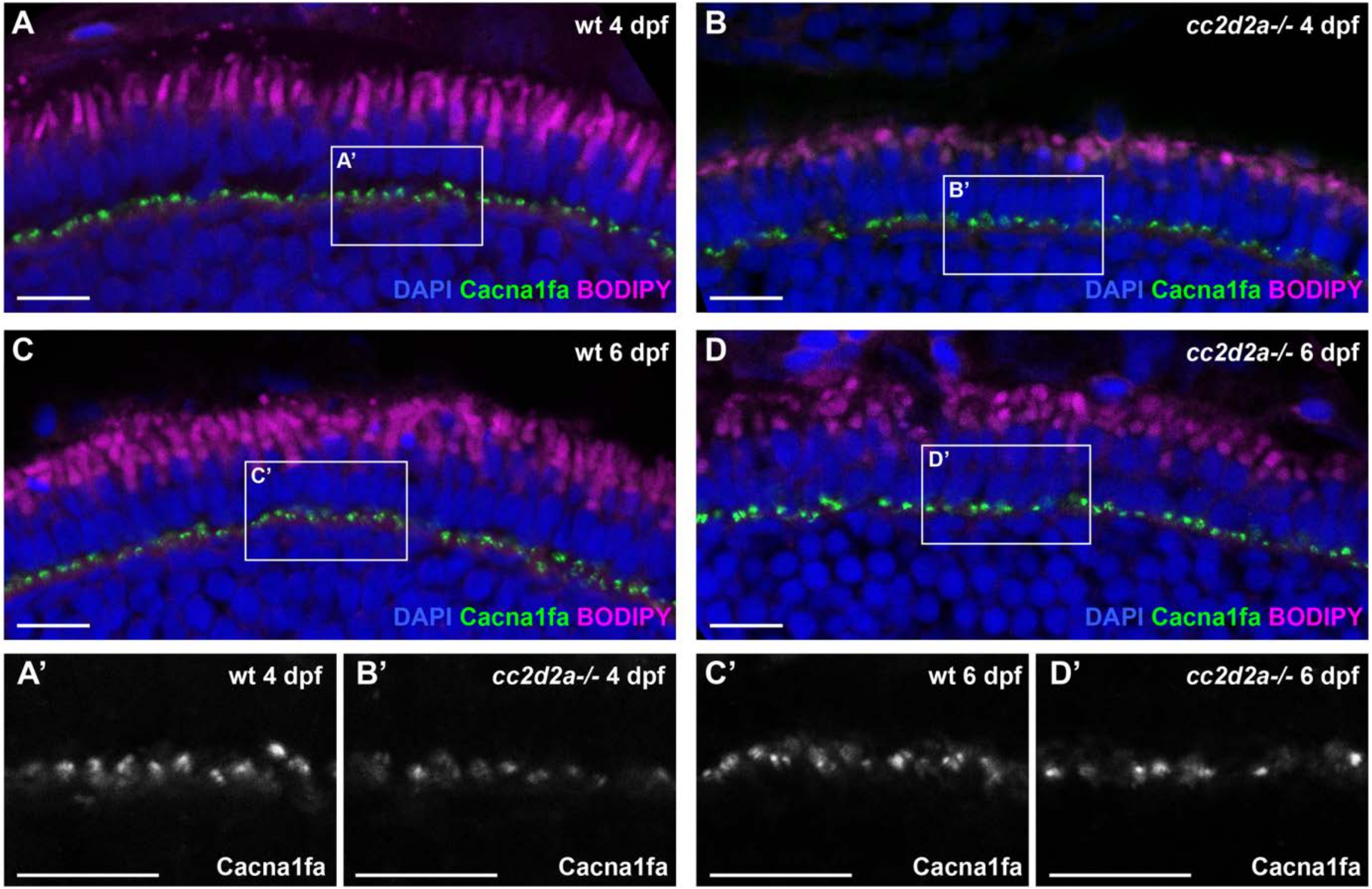

Supplement: S4 Fig — Retinal cryosections of 4 dpf (A-B) and 6 dpf (C-D) wild-type (wt) (A, C) and cc2d2a-/- (B, D) zebrafish, stained with an antibody against Cacna1fa (green), an L-type calcium channel that localizes to the synapse, and counterstained with DAPI (nuclei, blue) and BODIPY (outer segments and mitochondrial cluster, magenta). (A’-D’) Close-ups of the synaptic regions boxed in A-D. Cacna1fa (grey) in the mutant (B’ and D’) presents a similar localization at synapses as in wt (A’ and C’) at 4 and 6 dpf. Scale bars: 10 μm in all figures. (PDF) [file pgen.1007150.s012.pdf]
