# Supplementary material for: Loss-of-function of the ciliopathy protein Cc2d2a disorganizes the vesicle fusion machinery at the periciliary membrane and indirectly affects Rab8-trafficking in zebrafish photoreceptors
Source: PLoS Genet. 2017 Dec 27;13(12):e1007150. doi: 10.1371/journal.pgen.1007150 (PMC5760100; doi:10.1371/journal.pgen.1007150)

S11 Fig. Comparison of *cc2d2a* and *ift88* mutant retinae indicates that vesicle accumulation in PRs is not a general non-specific defect secondary to any ciliary dysfunction

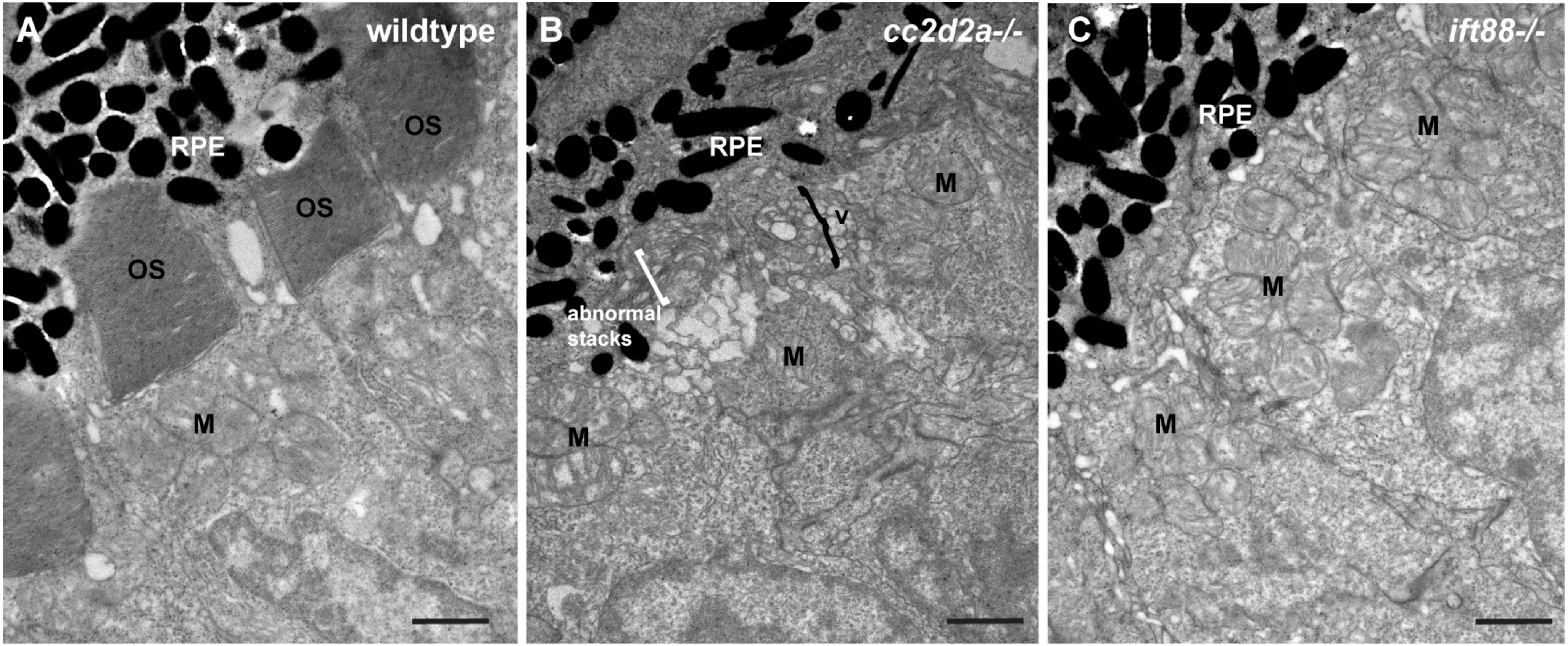

Supplement: S11 Fig — (A-C) Transmission electron microscopy images of 3 dpf wild-type (A), cc2d2a mutant (B) and ift88 mutant (C) retinae. Note the accumulation of vesicular structures and abnormal membrane stacks in cc2d2a mutants, while no vesicles are found in the inner segments of ift88 mutants. (PDF) [file pgen.1007150.s019.pdf]
